# Genomics of *Clostridium taeniosporum*, an organism which forms endospores with ribbon-like appendages

**DOI:** 10.1371/journal.pone.0189673

**Published:** 2018-01-02

**Authors:** Joshua M. Cambridge, Alexandra L. Blinkova, Erick I. Salvador Rocha, Addys Bode Hernández, Maday Moreno, Edwin Ginés-Candelaria, Benjamin M. Goetz, Scott Hunicke-Smith, Ed Satterwhite, Haley O. Tucker, James R. Walker

**Affiliations:** 1 Department of Molecular Biosciences and Institute for Cell and Molecular Biology, University of Texas, Austin, TX, United States of America; 2 Department of Natural Sciences, Health & Wellness, Miami Dade College-Wolfson Campus, Miami, FL, United States of America; 3 Center for Computational Biology and Bioinformatics, University of Texas, Austin, TX, United States of America; 4 Genomic Sequencing and Analysis Facility, Institute for Cell and Molecular Biology, University of Texas, Austin, TX, United States of America; Illinois Institute of Technology, UNITED STATES

## Abstract

*Clostridium taeniosporum*, a non-pathogenic anaerobe closely related to the *C*. *botulinum* Group II members, was isolated from Crimean lake silt about 60 years ago. Its endospores are surrounded by an encasement layer which forms a trunk at one spore pole to which about 12–14 large, ribbon-like appendages are attached. The genome consists of one 3,264,813 bp, circular chromosome (with 26.6% GC) and three plasmids. The chromosome contains 2,892 potential protein coding sequences: 2,124 have specific functions, 147 have general functions, 228 are conserved but without known function and 393 are hypothetical based on the fact that no statistically significant orthologs were found. The chromosome also contains 101 genes for stable RNAs, including 7 rRNA clusters. Over 84% of the protein coding sequences and 96% of the stable RNA coding regions are oriented in the same direction as replication. The three known appendage genes are located within a single cluster with five other genes, the protein products of which are closely related, in terms of sequence, to the known appendage proteins. The relatedness of the deduced protein products suggests that all or some of the closely related genes might code for minor appendage proteins or assembly factors. The appendage genes might be unique among the known clostridia; no statistically significant orthologs were found within other clostridial genomes for which sequence data are available. The *C*. *taeniosporum* chromosome contains two functional prophages, one *Siphoviridae* and one *Myoviridae*, and one defective prophage. Three plasmids of 5.9, 69.7 and 163.1 Kbp are present. These data are expected to contribute to future studies of developmental, structural and evolutionary biology and to potential industrial applications of this organism.

## Introduction

Bacterial endospore appendages are both common and highly diverse in structure, including ribbons, pili, feathers, brushes, tubules and swords (reviewed in [1, 2]). Moreover, their formation is highly variable even among closely related organisms. Different strains of the same species might or might not form appendages and different structural types can be formed by different strains of the same species [1]. Of special interest are the spore appendages of *Clostridium taeniosporum*. This organism, a Gram-positive, non-pathogenic anaerobe isolated from Crimean lake silt, is unique because its spores are surrounded by a thick "encasement" layer which forms a trunk at one spore pole from which about 12–14, large, flat, ribbon-like appendages emanate [3, 4, 5, 6]. The appendages—about 4.5 μm in length, 0.50 μm in width and 30 nm thick–are composed of smaller tennis-racket-like complexes (fibrils) (heads about 5 nm in diameter attached to tails about 1–2 nm in diameter and 40 nm in length) arranged in parallel rows with the heads forming one surface of the appendage [4, 6]. The smaller complexes are composed primarily of three proteins, two molecules of nearly identical 29 kDa paralogs and one molecule of a 37 kDa glycoprotein [6]. The 29 kDa proteins are thought to form the heads and the glycoprotein, which contains a collagen-like domain, is thought to fold back on itself into a triple-stranded, right-handed cylinder to form the tails [7, 8, 9, 10]. (The apparent difference between the 30-nm thickness of the appendages and the 40-nm length of the fibril tails is likely the result of different methods of preparation or bending of the fibril tails in the appendages.) Synthesized late in sporulation, the ribbons are coiled into a stalk-like structure attached to the spore pole near the mother cell mid-point and are so large that the stalk occupies most of the mother cell interior [4]. Each ribbon contains about 50,000 to 100,000 complexes and the complete set of appendages is assembled from about 600,000 to 1,200,000 molecules of each of the principal component proteins.

This organism is interesting for many reasons. First, study of the appendage function might contribute to microbial ecology. Perhaps the appendages serve as adhesive organelles to maintain spores in favorable anaerobic environments or perhaps to facilitate dispersal into new habitats. Second, fibril and appendage assembly studies should contribute to structural biology. Third, developmental biology problems of how appendages are positioned on one spore pole and how their size and shape are determined might be approached. Fourth, the evolutionary relationships of the non-toxigenic *C*. *taeniosporum* to its closest relatives, the neurotoxigenic *C*. *botulinum* Group II members [11], should be instructive. Fifth, the potential use of spores or purified appendages as surface display hosts in vaccine production, for drug delivery into hypoxic environments, and in nanobiotechnological applications should be explored. Finally, Gonchikov [12] has proposed that eukaryotic cells could have arisen from a clostridial cell which forms spores with ribbon-like appendages engulfing a euryarchaeon in an endosymbiotic process. To provide the basis for study of these and other interesting problems, the genome of *C*. *taeniosporum* has been sequenced and annotated.

## Results and discussion

### Genome general features

**Fig 1:**
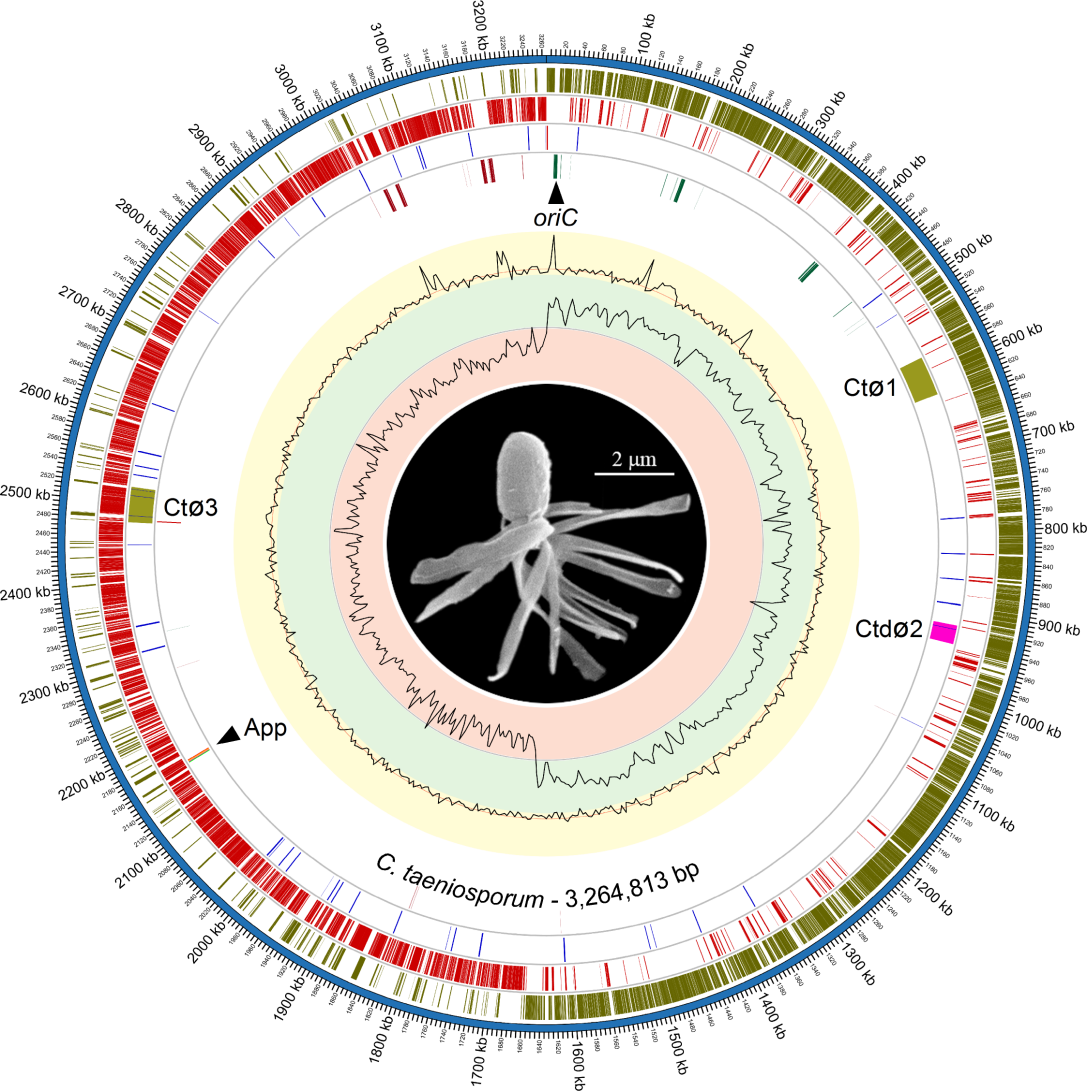
The *C*. *taeniosporum* spore and chromosome. The spore was observed by scanning electron microscopy as described [6]; the background was blackened by Photoshop. Photographs of other spores have been published [6, 11]. From the outside, circle 1 represents the chromosome in Kbp. Circles 2 and 3 represent potential protein coding sequences transcribed clockwise and counterclockwise in shades of green and red, respectively. The different shades of green and of red are assigned randomly to coding sequences, therefore, some adjacent, but otherwise unrelated, coding sequences have the same shades. Circle 4 includes blue and orange bars representing genes related to mobile elements transcribed clockwise and counterclockwise, respectively. Circle 4 also includes three prophages (labeled CtØ1, CtdØ2 (defective) and CtØ3). Circle 5 contains green and red bars to represent rRNA and tRNA genes transcribed clockwise and counterclockwise, respectively. Circle 6 (yellow background) represents GC percentage; the outermost and innermost edges of the yellow circle represent 50 and 20% GC, respectively; the red line is the *C*. *taeniosporum* average, 26.6%. Circle 7 (green and red backgrounds) shows GC skew [(G-C)/(G+C)] from +0.55 (outermost edge of green circle) to -0.55 (innermost edge of red circle). Locations of *oriC* and the spore appendage genes (App) are indicated. The map was generated by Circos 0.56 [13]. Bp 1 is the first bp of the first DnaA box of *oriC*. GC percentage was plotted every 5,000 bp; GC skew was measured over 10,000 bp windows resampled every 5,000 bp. CpG Islands 1.1 did not detect genomic islands.

The *C*. *taeniosporum* chromosome is a circle of 3,264,813 bp (Fig 1) with a total of 2,892 potential protein coding regions covering 84.03% of the chromosome. Of these, 2,271 can be assigned specific (2,124) or general (147) functions (Table 1. The remaining 621 have unknown functions, of which 393 are hypothetical genes, based on the fact that database searches did not reveal a match with a cutoff *E* value of 10^−5^ or less [14], and might be unique to *C*. *taeniosporum*. A total of 62 genes encode transposases (10 in the IS256 family [15]) or other proteins related to mobile elements (S1 Table) and are included in the Table 1 Replication/Repair/Recombination functional category. Included also are 101 stable RNA genes—seven rRNA gene clusters and 78 tRNA genes (S2 Table). Although some clostridia have selenocysteine tRNA genes [16], *C*. *taeniosporum* apparently has neither the tRNA-Sec gene nor the *sel* operon (discussed below). The chromosome is composed of 26.6% GC, typical of clostridia [17, 18], with tight distribution around the average, except for the seven ribosomal RNA gene clusters in which the GC percentage is markedly higher. The putative origin of replication, *oriC*, identified by (1) the similarity of its sequence to origins of other Gram-positive bacteria, (2) GC skew and (3) the direction of transcription of individual genes [19, 20, 21, 22], is proposed to consist of two untranslated DnaA Box clusters bracketing the *dnaA* gene. A similar region of the *Bacillus subtilis* chromosome, even with the central *dnaA* gene deleted, is an autonomous replicating sequence [23]. Bacterial leading strands often contain more G's than C's, a fact which is useful in identifying origins and termini [24, 25]. *C*. *taeniosporum* replichores 1 and 2 are clearly marked by almost entirely positive and negative values with averages of +0.254 and -0.238, respectively (Fig 1). Replichore 1, replicated clockwise, is also transcribed predominantly clockwise (87.3% of the CDSes); replichore 2, replicated counterclockwise, is also transcribed predominantly counterclockwise (81.7% of the CDSes). All seven ribosomal RNA gene clusters and 74 of the 78 tRNA genes are also oriented with the replication direction. This preferential orientation of genes with the replication direction [26, 27, 28] has the advantage of avoiding head-on collisions of replication and transcription complexes [29, 30]. Single copies of the appendage genes are located in one cluster. Three prophages are located within the chromosome and three extrachromosomal plasmids totaling 241.3 Kbp are present also. *C*. *taeniosporum* is among the relatively rare clostridia which neither synthesize selenoproteins nor incorporate selenium into 2-selenouridine in tRNAs [31].

**Table 1:** Functional categories of *C*. *taeniosporum* chromosomal protein CDSes.

Code	Functional Category	CDSes (number)
A	RNA processing/modification	ND
B	Chromatin structure/dynamics	7
C	Energy production/conversion	148
D	Cell cycle control/division	28
E	Amino acid metabolism/transport	164
F	Nucleotide metabolism/transport	104
G	Carbohydrate metabolism/transport	194
H	Coenzyme metabolism	90
I	Lipid metabolism	65
J	Translation	169
K	Transcription/control	168
L	Replication/repair/recombination	183
M	Cell wall/membrane/envelope	155
N	Cell motility/chemotaxis	94
O	Post-translational modification/protein turnover/chaperone	91
P	Inorganic ion metabolism/transport	100
Q	Secondary structure	1
R	General function prediction	147
S	Function unknown	
	Conserved	228
	Hypothetical	393
T	Signal transduction	70
U	Intracellular trafficking/secretion	24
V	Phage related	138
W	Sporulation/control/appendages	89
X	Drug resistance/bacterial toxins	42
**Total**		**2892**
ND, none detected		

### Replication origin

**Fig 2:**
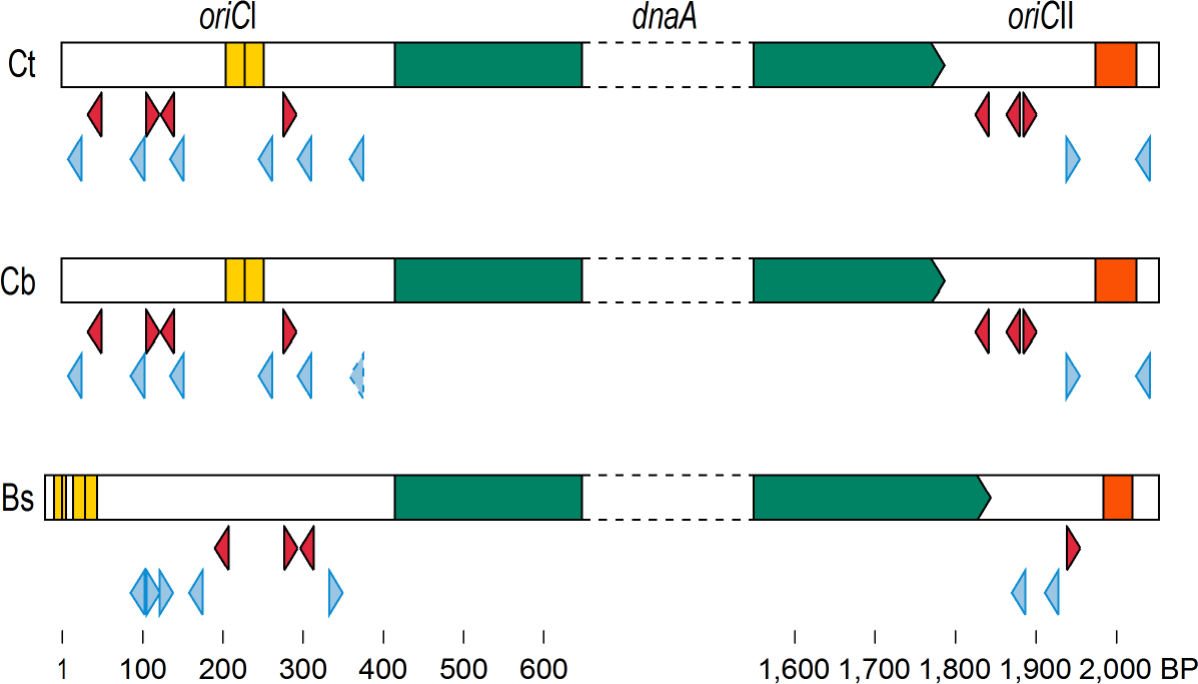
Replication origins of *C*. *taeniosporum* (Ct), *C*. *botulinum* (Cb), and *B*. *subtilis* (Bs). Untranslated, DnaA box-rich segments bracket the *dnaA* gene (green arrows indicate transcription direction). DnaA boxes which match the low G+C Gram positive bacteria consensus of TTATCCACA [32, 33] in all 9 (maroon triangles), in 8 (blue triangles) and in 7 (dashed blue triangle) positions. Direct repeats are indicated by yellow bars; a potential DNA Unwinding Element by the red bar. *C*. *botulinum* B strain Eklund 17B (NC_010674.1) and *B*. *subtilis* strain 168 (NZ_CM000487.1) data were used. The nucleotides are numbered beginning with the first nucleotide of the *C*. *taeniosporum oriC* region.

The putative *oriC* was identified by the orientation of genes in two replichores, base composition asymmetry, the presence of DnaA boxes and the *dnaA* gene and the locations of genes frequently found near known origins [19]. *oriC* is on a 9.4 Kbp region which contains *rnpA*, *rmpH*, *oriC*I, *dnaA*, *oriC*II, *dnaN*, *recF*, *orf*68, *recF*, *orf*87, *gyrB* and *gyrA*, similar to the gene organization of the origins of Gram-positive organisms [20, 21, 22]. *oriC* is proposed to consist of two untranslated DnaA box clusters bracketing the *dnaA* gene. *oriC*I is an untranslated 420 bp sequence containing ten putative DnaA binding sites which match the consensus (TTATCCACA for low G+C Gram positive *Firmicutes*) [32, 33] in at least 8 of the 9 positions and also two direct repeats (Fig 2). *oriC*II is also untranslated and consists of 234 bp containing five DnaA boxes (at least 8 matches to the consensus) and an AT-rich, potential DNA Unwinding Element (50 AT pairs within a 53 bp region) near the 3’ end (Fig 2). The presence of direct repeats and the DNA Unwinding Element is also characteristic of origins. The nucleotide sequence is very similar to that of the closest relative, *C*. *botulinum* B strain Eklund 17B (GenBank Accession NC_010674), except that one *oriC*I DnaA box in the latter organism matches the consensus in 7, rather than 8, positions (Fig 2). Although the oriCII region alone is capable of autonomous replication in some organisms [34, 35, 36], autonomous replication of an *oriC* plasmid in *B*. *subtilis* requires both *oriC*I and *oriC*II, but not the *dnaA* gene itself [23].

### Sporulation in the clostridia

The overall process of forming spores under control of the sigma cascade started by phosphorylated Spo0A is basically similar in *Bacillus subtilis* and in the clostridia, but there are many differences between sporulation in *B*. *subtilis* and the clostridia especially in the control, as reviewed recently by Al-Hinai et al. [37]. The clostridia are a very diverse group. Collins et al. [38] described about twenty clusters of the clostridia and Yutin and Galperin [39] have proposed that the *Clostridia* should include also the Negativicutes (Gram positive bacilli which form spores and have evolved to form also Gram negative envelopes and phenotypes). Even within the *C*. *botulinum* species, there are four groups; the members of each group are closely related to each other, but distantly related to members of the other three groups [40]. *C*. *taeniosporum* is a non-toxigenic member of the *C*. *botulinum* Group II [41]. Given such diversity, it is not surprising that major patterns of controlling spore formation differ within the clostridia and between clostridia and *B*. *subtilis*.

First, nutrient deprivation is the signal to sporulate in *B*. *subtilis* and in some clostridia [37], but in the solventogenic clostridia, the accumulation of organic acids and lower pH are thought to initiate spore formation even in the presence of excess nutrients [42]. Second, the first observable morphological change in clostridia is a shift from uniform bacilli to the swollen, rounded clostridial cell form [43], a form not observed in *Bacillus* [37]. A third major difference between *Bacillus* and the clostridia is the mechanism of Spo0A activation. In *B*. *subtilis*, histidine kinases phosphorylate phosphorelay proteins which transfer the phosphate to Spo0A to start the sporulation sigma cascade (σ^F^, σ^E^, σ^G^, σ^K^) [37]. In the clostridia, Spo0A is phosphorylated directly by orphan histidine kinases (i.e., those without cognate response regulators) without participation of phosphorelay proteins. Moreover, the details of the Spo0A activation differ among the different clostridial species in the number and identity of the histidine kinases [37]. Fourth is the role of σ^K^. In *B*. *subtilis*, σ^K^ functions late in the mother-cell [44]. In several clostridial species, σ^K^ functions both late in the mother cell and also prior to stage II (asymmetric septation) [37]. It is required for Spo0A synthesis in *C*. *acetobutylicum* and at least one strain of *C*. *botulinum* [45, 46]. Finally, the control of expression of the σ^H^ gene appears to differ between *B*. *subtilis* and at least one *Clostridium*. In the former, σ^H^ is involved in the transition from exponential to stationary phase [47] and in the expression of a histidine kinase which initiates the Spo0A phosphorylation pathway [48]. σ^H^ expression, from both σ^A^ and σ^H^-dependent promoters, is then up-regulated by activated Spo0A [49]. In *C*. *acetobutylicum*, the *sigH* gene is expressed from a σ^A^-dependent promoter and its expression level is higher throughout the culture cycle than that of the general transcription sigma A [50]. Finally, the nature of spore appendages varies from species to species in both genera. In *C*. *taeniosporum*, sporulation begins even in the presence of excess nutrients, making it typical of most clostridia. At least 89 *C*. *taeniosporum* genes code for spore components, including appendage proteins, or for regulatory factors.

### Appendage genes and proteins

**Fig 3:**
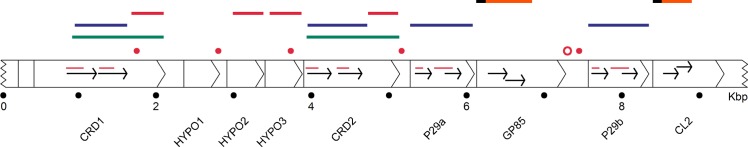
Appendage genes and proteins. Appendage protein genes P29a, P29b and GP85 and related genes (open arrows) reside on a 9.6 Kbp region of the chromosome and are transcribed in the same direction (arrow points). Extensive homology of the deduced proteins (or regions therein) is indicated by colored bars above the relevant portions of the genes: CRD1 and CRD2 (green), portions of CRD1 and CRD2 with most of P29a and P29b (blue), portions of CRD1 and CRD2 with most of the HYPO2 and HYPO3 proteins (red) and portions of GP85 and CL2 are identical (black) and both have collagen-like regions (orange). Internal repeats and DUF11 sequences within deduced protein sequences are indicated by black arrows and red bars, respectively. Positions of potential mother cell late promoters (red circle) and a potential sigma A-dependent promoter (open red circle) are indicated. The filled circles indicate Kbp positions. Bp 1 corresponds to the complement of chromosomal nucleotide pair 2,172,100.

The spore appendages, composed of small, tennis racket-like fibrils arranged in parallel rows [3, 4, 5, 6], are composed principally of three proteins, two 29 kDa isoforms designated P29a and P29b and a glycoprotein of 37.5 kDa (deglycosylated) containing a collagen-like region and designated GP85 [6]. The genes for these and closely related proteins are single copy, adjacent, located on a 9.6 Kbp region of the chromosome in the order: CRD1, HYPO1, HYPO2, HYPO3, CRD2, P29a, GP85, P29b, and CL2 (Fig 3) and are all transcribed in the same direction. The CRD symbols indicate that the (deduced) protein products are conserved and contain repeat domains but without known functions. The HYPO symbols indicate the genes are hypothetical coding sequences without significant homology to known proteins. The CL2 symbol indicates that the deduced protein also contains a collagen-like region.

All the deduced protein products of these genes, with the exception of HYPO1, are highly related. First, many of them share extensive sequence similarity (Fig 3; S3 Table) with one or more other proteins of this group. CRD1 and CRD2 (564 and 413 residues, respectively) share 399-residue regions which are 36.6% identical and 68.7% similar. Both P29a and P29b contain 269 residues, 87% of which are identical. Moreover, P29a and P29b share extensive similarity to both CRD1 and CRD2; 225 residues of P29a and P29b (of the 269 residue total) are about 22% identical to a similar region of the CRD1 protein and 258 of their residues are about 29% identical to a similar region of the CRD2 protein. HYPO2 and HYPO3 (158 and 160 residues, respectively) are 34% identical and 65% similar over 140 residues, which constitute most of their lengths, and both share extensive similarity to regions near the C-termini of both the CRD1 and CRD2 proteins. Finally, the GP85 and CL2 proteins are identical over the first 39 residues and collagen-like regions cover 239 and 129 residues in GP85 and CL2, respectively.

Second, six of the nine proteins contain internal repeats, ranging from the shortest 65- to the longest 127-residue repeats in the CL2 and CRD1 proteins, respectively (Fig 3; S3 Table). Third, four of the nine proteins contain the domain of unknown function, DUF11, within repeat regions. This conserved domain [51, 52] (http://www.ebi.ac.uk/interpro/entry/IPR001434) contains about 76 residues and is present in cell envelope proteins, often within internal repeats, of unknown function in a wide range of distantly related prokaryotes, including Archaea. Examples include three spore proteins of the *Bacillus cereus* group (CrdA, CrdB and CrdC) [53], a *Chlamydia trachomatis* major outer membrane complex protein [54] and an archaeal *Methanosarcina mazei* cell surface protein [55].

Fourth, collagen-like regions are present in two proteins. Collagens form connective tissues in higher organisms and contain left-handed helices of repeating GXY sequences which wind around a central axis forming right-handed, triple helical, rod-like structures [7, 8]. The GXY repeats often include proline and hydroxyproline as the X and Y residues in higher organisms. Some bacterial and phage structural proteins also contain collagen-like regions of GXY repeats which form stable triple helices, although they lack hydroxyproline [56]. The surface proteins of *Streptococcus pyogenes* Scl1 and 2 have lollipop shapes with the collagenous regions folding back on themselves to form the rods [56]. The *Bacillus anthracis* exosporium BclA protein contains a collagen-like region and is similar in shape to the *C*. *taeniosporum* appendage fibrils [57]. Some phage structural components also include proteins with collagen-like regions [58, 59].

The appendage genes are highly expressed; each cell must synthesize at least 600,000 molecules of each P29a, P29b and GP85 late in sporulation to assemble about 50,000 fibrils for each of the 12 appendages. There are five strong candidates for late mother cell sigma K-dependent promoters in the appendage gene region. All five match the consensus sigma K promoter sequence [a/cACa/c N16 CATA N3 TA] [60, 61] perfectly (or with one mismatch), all have the consensus spacing and all contain the most highly conserved AC of the -35 region (Fig 3).

Two of the putative sigma K promoters are located in the intergenic regions upstream of the P29a - GP85 and the P29b - CL2 genes and these pairs likely form operons. The putative sigma K promoter upstream of the P29a gene matches the consensus perfectly and the one upstream of the P29b gene has only one mismatch. A putative sigma A-dependent promoter upstream of the P29b and CL2 genes suggests that this operon is expressed in vegetative growth, as well as late in sporulation. Additionally, three putative sigma K promoters are located within the CRD1, HYPO1 and HYPO3 reading frames (Fig 3). Although promoters usually are located within intergenic regions, sigma A-dependent promoters can be found within reading frames [62]. Two well characterized, low level, constitutively expressed promoters are located within the *Escherichia coli trp* and *ilv* reading frames [63, 64]. Additional sequences which match the sigma K consensus with two or three mismatches are located within this appendage gene region, but have not been labeled as putative promoters.

### Prophages

*C*. *taeniosporum* contains two complete and one defective prophages. Prophage CtØ1 consists of 37,424 bp, thirty-six potential coding sequences (10 of which code for hypothetical proteins, mostly conserved among phage proteins) and putative attachment sites (S4 Table). Its GC content is 30.19%. The second prophage, CtdØ2, is likely to be defective; no attachment sites, phage head protein genes or site-specific integrase genes were detected. It consists of only 22,430 bp with 26.26% GC content. Among the 27 potential coding sequences, 10 code for hypothetical, but mostly conserved, proteins (S5 Table). The third prophage, CtØ3, has 28.6% GC and consists of 43,915 bp, 54 potential coding sequences (28 hypothetical, mostly conserved) and potential attachment sites (S6 Table). All the prophage orfs are listed in the Phage-related functional category in Table **1** and the prophage orfs related to recombination/integration are listed also in S1 Table (Genes associated with mobile genetic elements).

**Fig 4:**
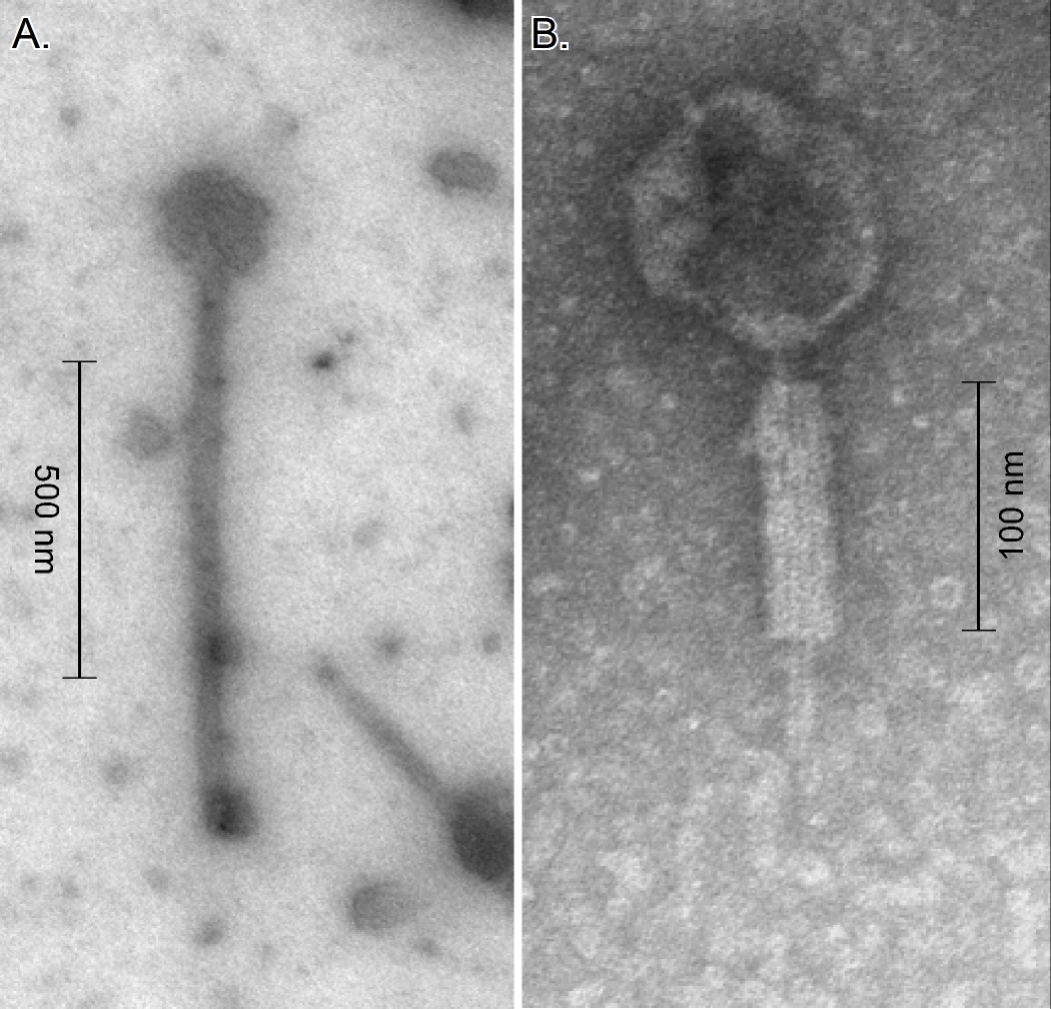
***C*. *taeniosporum* phages CtØ3 (*Siphoviridae*) (A) and CtØ1 (*Myoviridae*) (B).** Phage particles concentrated from culture supernatant were observed by transmission electron microscopy.

Both the CtØ1 and CtØ3 prophages are functional. After incubation of *C*. *taeniosporum* in medium containing mitomycin C, phage particles were observed by electron microscopy in the concentrated culture fluid (Fig 4). The phage observed in greater numbers has a longer, flexible, non-contractile tail (typical of the *Siphoviridae*); the phage with a shorter, contractile tail was observed much less frequently and is typical of the *Myoviridae* [65, 66]. Based on the number of nucleotides in the tail tape measure protein genes, CtØ3 is likely to be the *Siphoviridae*; CtØ1 the *Myoviridae* [67, 68]. PCR amplification and sequencing of DNA fragments from the concentrated phage particles confirmed the presence of CtØ3 sequences.

**Fig 5:**
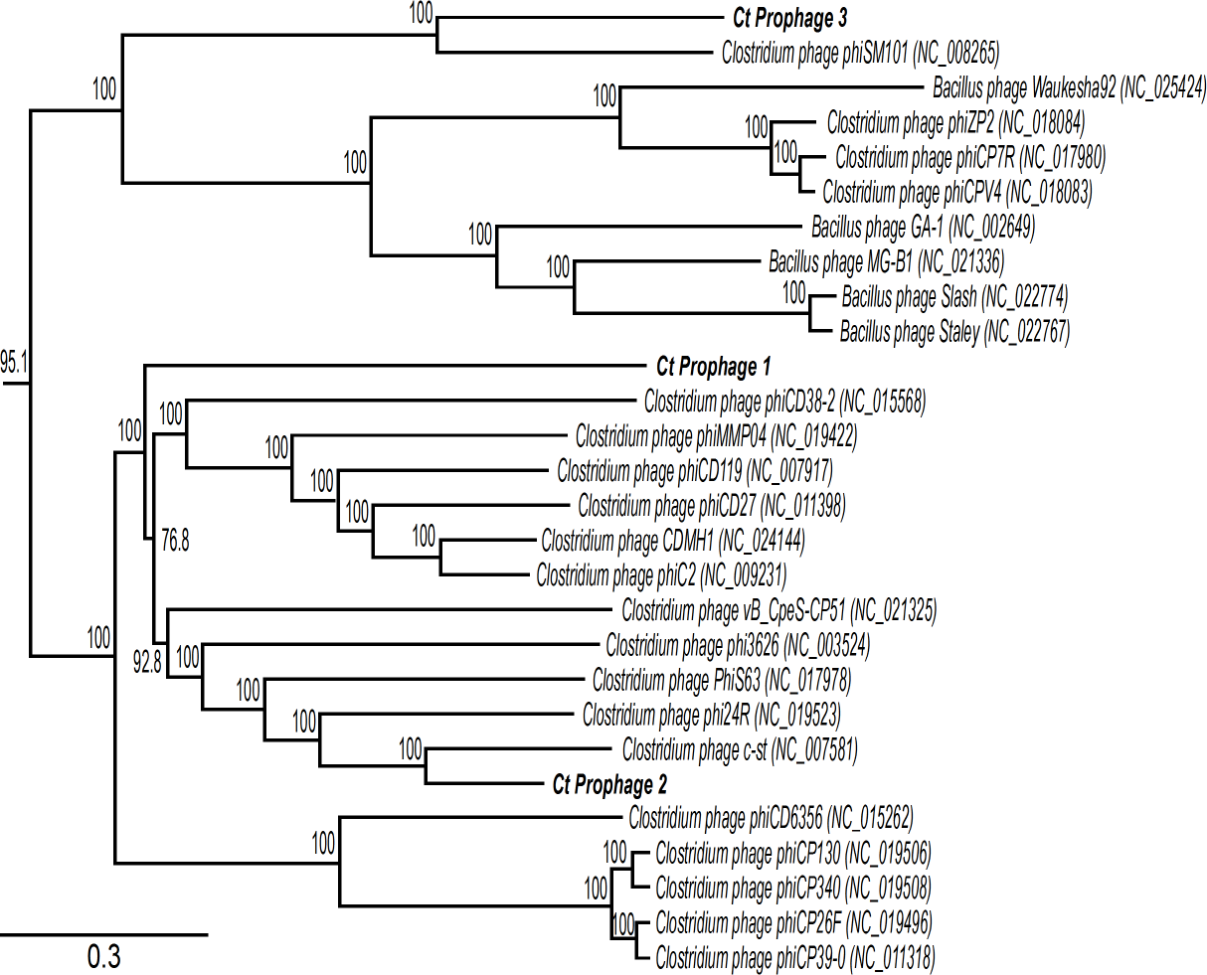
Neighbor-joining phylogenetic tree of a selected cluster of the phages which infect the *Firmicute Clostridium* and *Bacillus* genera. The nucleotide sequences of the 62 phages known to infect the *Clostridium* or *Bacillus* genera (and for which complete nucleotides sequences were available) were subjected a MAFFT multi-wise alignment [69]. A neighbor-joining tree [70] was constructed (Geneious Pro v.7.1.6) and bootstrap values, expressed in percentage based on 1,000 repetitions, are shown next to each group. The bar represents 0.3 change per nucleotide site.

All three prophages are closely related to known clostridial and bacillus phages (Fig 5). CtØ1 and CtdØ2 are very similar to a clade of 16 clostridial phages; CtØ1 is most closely related to *Clostridium* phage phiCD38-2 (NC_015568); they are 59.2% identical in more than 37 Kbp of sequence. The defective, 22 Kbp CtdØ2 is most closely related to a portion of the 185.7 Kbp *Clostridium* phage c-st (NC_007581); it is 54.7% identical to the 9.5 to 31.5 Kbp region of the c-st phage. CtØ3 is most closely related to the *Clostridium* phage SM101 (NC_008265); they share 52.8% identity over 39.9 kb.

### Selenium metabolism

Selenium has three major activities in prokaryotes, incorporation into selenoproteins, incorporation into 2-selenouridine-containing tRNAs, and action as a cofactor in certain molybdenum-containing hydroxylases. The first two of these functions depend on the synthesis of selenophosphate by selenophosphate synthetase, the *selD* gene product [31, 71]. The selenoproteins, common among anaerobes, contain selenocysteine and are required for growth in amino acid media to catalyze Stickland reactions and harvest metabolic energy by the coupled anaerobic oxidation and reduction of amino acid pairs resulting in ATP production by substrate level phosphorylation [72, 73, 74, 75]. The incorporation of selenocysteine into protein requires the products of the *selA*, *selB* and *selC* genes (reviewed in Böck [76]), which are not present in *C*. *taeniosporum*. The replacement of sulfur in 2-thiouridine in the tRNAs which contain that modification [77] requires 2-selenouridine synthase, the product of the *ybbB* gene [78], also apparently missing from the *C*. *taeniosporum* genome.

Therefore, this organism is among the relatively rare SelD orphans [31]–organisms which have the *selD* gene and presumably synthesize selenophosphate by action of selenophosphate synthetase, but neither synthesize selenoproteins nor incorporate selenium into 2-selenouridine in tRNAs. SelD orphans account for about 5% of SelD-containing organisms [31]. This raises two questions. First is the function of selenophosphate synthetase in SelD orphans? Selenium is a labile cofactor in some molybdenum-containing hydroxylases, including xanthine dehydrogenase [79, 80, 81] and a purine hydroxylase [82], although the structure of the Se is apparently not known [31]. *C*. *taeniosporum* contains two copies of a Se-dependent xanthine dehydrogenase (*xdh*) gene and at least four other genes coding for Se metabolism-linked proteins. Perhaps, SelD is required for incorporation of Se as a labile cofactor in these or other proteins. Second is the energy harvesting mechanism of the anaerobe in the absence of selenoproteins to catalyze Stickland reactions. Perhaps some oxidoreductases involved in energy metabolism also use non-covalently linked Se as a cofactor. For example, the selenium- and molybdenum-containing nicotinic acid hydroxylase of *Clostridium barkeri* requires a labile form of Se which is directly coordinated with molybdenum [83, 84].

### *C*. *taeniosporum* plasmids

**Fig 6:**
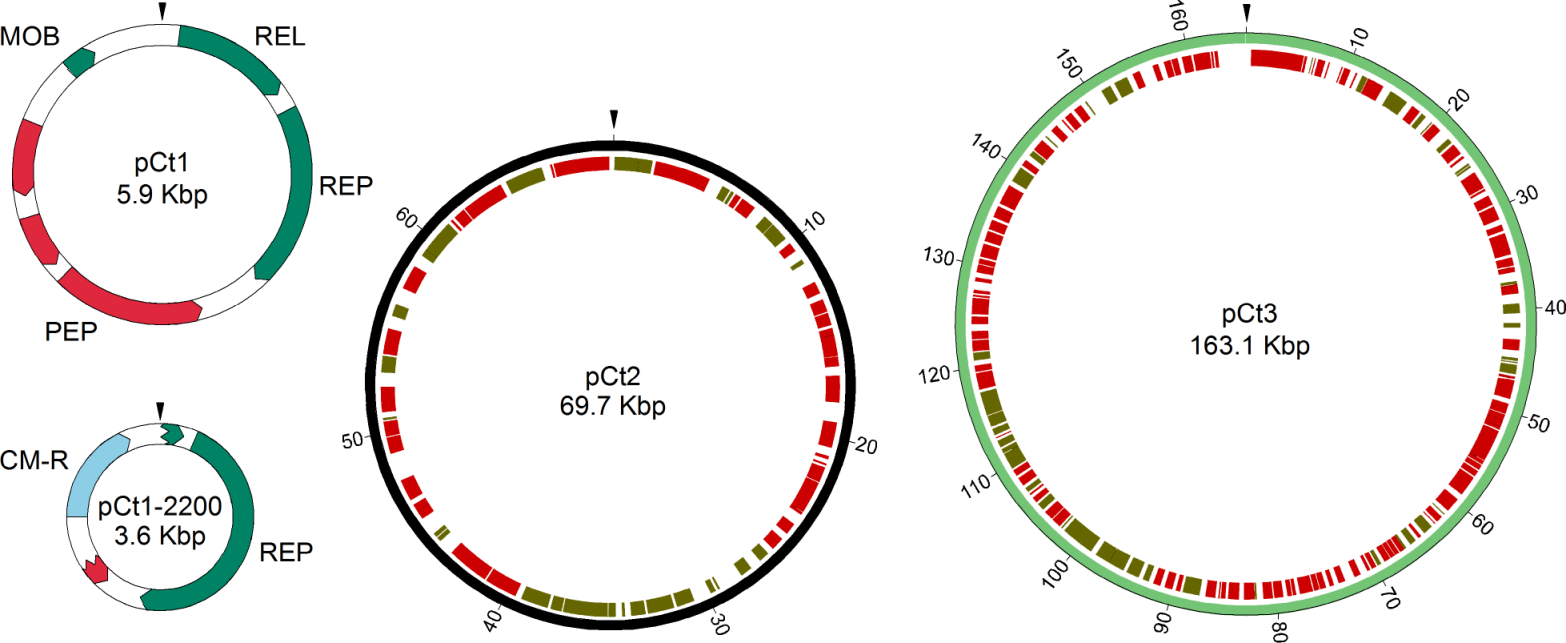
Naturally occurring plasmids of *C*. *taeniosporum* and the derivative pCt1-2200. Locations and transcription directions of genes of pCt1 and pCt1-2200 are indicated by arrows. pCt1 genes include those for mobilization (MOB), relaxase (REL), plasmid replication (REP) and peptidase (PEP) proteins. The replicon of pCt1 (present within a fragment consisting of a portion of the REL gene, the replication gene, the intergenic region and a portion of the PEP gene) was ligated to a chloramphenicol-resistance (CM-R) fragment of pAT4, generating pCt1-2200 which replicates in *B*. *subtilis*. pCt2 and pCt3 consist of 62 and 154 potential coding sequences; transcription direction clockwise (green) or counterclockwise (red). The arrow points mark bp 1.

*C*. *taeniosporum* contains three plasmids: pCt1, pCt2 and pCt3. pCt1 consists of 5,894 bp and contains genes for a replication protein, a relaxase, a mobilization protein (MobC homolog) and three genes of unknown function (some contain conserved domains) (Fig 6; S7 Table). The putative replication protein is highly similar in sequence to nine plasmid and chromosomal replication factors of a wide range of organisms, including other clostridia, *Geobacillus*, *Pseudomonas*, *Aeromonas* and *Yersinia*. It is most closely related to the putative replication proteins of the *Clostridium perfringens* plasmid pSM101B (YP_699929; NC_008264) and of the host *Clostridium perfringens* SM101 chromosome (YP_697960; NC_008262 [85]) (69% identical over 399 residues to the plasmid protein and 69% identical over 335 residues to the chromosomal gene protein) [85]. It is 42% identical over 395 residues to the *Geobacillus stearothermophilus* plasmid pGS18 putative replication protein (YP_001716004; NC_010420 [86]). The presence of the potential relaxase and MobC genes might indicate that this plasmid could be mobilized for conjugal transfer.

The pCt1 replicon, present on the 2.4 Kbp fragment containing the 3’ end of the relaxase, the replication protein gene *rep*, the intergenic region downstream of *rep* and a portion of the peptidase gene, is sufficient for replication in *Bacillus subtilis*. Plasmid pCt1-2200, which consists of that 2.4 Kbp pCt1 replicon linked to a 1.2 Kbp chloramphenicol-resistance (CM-R) determinant from pAT4, transformed *B*. *subtilis* to chloramphenicol-resistance. pCt1-2200 was extracted from the *B*. *subtilis* transformants and its structure confirmed. Therefore, the replication gene and the adjacent nucleotides also contain the plasmid replication origin.

pCt2 consists of 69,744 bp and 62 potential orfs, many of which code for likely useful proteins. Genes of a type I restriction/modification system [87] include those for two M and two S subunits; all located within an 8.2 Kbp region. A cytosine-specific DNA methylase gene is present; this enzyme could be involved also in restriction/modification. Four replication genes are present on this plasmid. One likely codes for a plasmid replication factor; the deduced protein is 32% identical over more than 400 residues to the pCt1 replication protein and more than 80% identical (over 437 residues) to five *C*. *beijerinckii* and *C*. *botulinum* replication proteins. Other close relatives include the *Geobacillus stearothermophilus* pGS18 and the *C*. *perfringens* pSM101B plasmid replication proteins and the replication protein encoded by the *C*. *perfringens* strain SM101 chromosome. The other three copies of replication genes on pCt2 are significantly similar to the chromosomal *dnaD*; two of the DnaD proteins are 59% identical over 403 residues to DnaD encoded by the chromosome. DnaD is required for DNA replication initiation and re-initiation in *B*. *subtilis* and probably also in other low GC Gram-positive organisms [88]. Other potentially useful pCt2 genes include those which code for bacteriocin synthesis and immunity, quorum sensing, signal transduction proteins, a sigma 70 gene, Soj (a sporulation initiation inhibitor), transporter/antiporter pairs and three putative drug resistance determinants (kanamycin, bacitracin and a multidrug MATE transporter). A putative altruism determinant is present on this plasmid. The abortive infection (Abi) protein is highly related (E-value 7.0e-113) to known factors which, after phage infection, stop progeny phage production but, in the process, kill the infected host, thereby protecting the un-infected cells [89].

pCt3 consists of 163,055 bp and 154 potential genes. Among these are five genes encoding thiamine biosynthesis enzymes on a 4.7 Kbp region, genes for iron and cadmium translocating systems, seven genes encoding transcription regulators, genes for a type III restriction/modification system [90], and five genes for potential drug resistance. pCt3 also contains two CRISPR-like regions. One consists of about 500 bp of nine identical 30-mers repeated directly, separating variable regions of 34–37 bp. The second consists of about 700 bp of thirteen 30-mers also repeated directly and separating variable regions of 34–37 bp. The direct repeats in the two regions differ in only one of 30 bp. Although orfs on both sides of both repeat regions could code for Cas proteins, none of them is orthologous to known Cas proteins (reviewed by Barrangou [91]). Of special interest are components of toxin-antitoxin systems [92, 93] which serve to stabilize the plasmid presence. Potential toxin genes include those for a Fic/DOC family protein [94, 95] and the Zeta protein [96]. There is also a potential prevent-host-death antitoxin gene [92, 93, 94].

### Firmicutes and the origin of eukaryotic cells

Theories on the formation of eukaryotic cells can be divided into two major groups, endosymbiotic and autogenous. The former supposes that the eukaryotic nucleus was formed from one prokaryotic cell incorporated by another thereby generating both nucleus and cytoplasm; the latter that differentiation of nucleus and cytoplasm occurred by stepwise changes within a single lineage (reviewed by Martin [97] and Baum [98]). Gonchikov [12] has proposed that a eukaryotic cell could have been formed from an anaerobic clostridial cell which formed spores with appendages and a euryarchaeon by an endosymbiotic process. In this model, the clostridial mother cell cytoplasmic membrane erroneously engulfed a euryarchaeon cell during the engulfment stage of sporulation; the euryarchaeon became the eukaryotic nucleus and the spore appendages became the microtubular mitotic apparatus.

## Conclusions

The *C*. *taeniosporum* genome consists of a single circular chromosome of 3.26 Mbp, including two prophages and one defective prophage, plus three plasmids and includes numerous genes which code for proteins related to mobile elements, all suggesting that this organism has undergone many genetic exchanges. The three known appendage protein genes are single copy, which is surprising given the huge number of protein molecules needed for assembly of all twelve appendages, and are located in one 9.6 Kbp region of the chromosome along with five other closely related protein genes. The relatedness of the proteins and the proximity of their genes suggest that all those gene products could be involved in appendage production and assembly. Structural and developmental biological studies of the appendages including the mechanism by which they are attached to one spore pole, should, indeed, be very informative. Although *C*. *taeniosporum* is thought to be nonpathogenic, it evolved from a common ancestor of the *C*. *botulinum* Group II members [11], suggesting that more detailed study of *C*. *botulinum* and *C*. *taeniosporum* phylogeny and ecology would be useful.

## Materials and methods

### Strains, culture conditions and plasmid

*C*. *taeniosporum* strain 1/k was grown in modified CDC anaerobe medium (tryptic soy broth (30 g/l), yeast extract (5 g/l), NaCl (5 g/l), hemin (5 mg/l), vitamin K1 (10 mg/l), and glucose (5 g/l), pH 7.4 with agar (1.5%), as needed) [99] at 30°C under an atmosphere of 85% nitrogen, 10% hydrogen and 5% carbon dioxide in a Forma model 1025 anaerobic chamber. All solutions were reduced for 24 hr before use. *Escherichia coli* strain JM109 was grown on LB medium modified to contain 5 g NaCl/l; *Bacillus subtilis* strain SMY [100] was grown on LB medium. Ampicillin was added to 100 μg/ml and chloramphenicol to 30 μg/ml for selecting drug-resistant transformants of *E*. *coli*, chloramphenicol-resistant transformants of *B*. *subtilis* were selected on LB medium with 5 μg chloramphenicol/ml. pAT4, constructed by Charles Stewart and provided by Mary Harrison, carries the chloramphenicol-resistance gene of pC194 (NC_002013) [101].

### Molecular techniques

Chromosomal DNA was extracted from cells growing exponentially by the Puregene Genomic DNA Purification kit (Gentra Systems, Minneapolis, MN). Plasmids were purified by a QIAprep Kit [QIAGEN Inc., Valencia, CA 91355]. PCR reactions were conducted in a MasterCycler Personal (Eppendorf AG, Hamburg, Germany). Reagents included Taq DNA polymerase from Roche (Branford, CT), deoxyribonucleotides from Sigma-Aldrich (St Louis, MO) for short sequences and the RangerMix from Bioline (Taunton, MA) for amplification of fragments longer than 6 kbp. Transformations were performed by standard methods [102, 103].

### Genome sequencing and assembly

The *C*. *taeniosporum* genome was initially sequenced with 454 Life Sciences (Branford, CT) technology and the reads assembled with the Roche Newbler assembler program version 2.7 (Branford, CT) into 104 large contigs (average length of 33,000 bp). These larger contigs were further assembled with paired-end data into 18 scaffolds with average length of 192,000 bp, although a total of 73 gaps remained within the scaffolds. Additional data to close the gaps and connect the scaffolds were obtained by PCR to generate fragments spanning the gaps and linking scaffold ends followed by Sanger sequencing [104]. The assembly editing program, Consed version 22.0 [105], using the Autofinisher parameter, designed 203 primers and suggested 94 potential pairings; sequencing the resulting products closed 58 of the 73 gaps. An additional 38 primer pairs were designed manually and used to amplify fragments, the sequences of which closed the remaining gaps within scaffolds and joined the 18 scaffolds into 4. The largest was the 3.26 mbp circular chromosome, the remaining 3 were circular plasmids.

The genome was then sequenced by the Illumina method (Illumina, Inc., San Diego, CA) and the reads assembled into contigs, as indicated above. The Illumina data and the Sanger sequences were mapped to the 454 sequence by Geneious to create a consensus. The discrepancies between the Illumina data and the 454 data were resolved in favor of the Illumina data which have a reduced error rate [106] except in those cases in which the discrepancies were in regions covered by both 454 and Sanger sequencing. In those cases, the 454/Sanger data were favored. Overall, there were 63 corrections to the 3.26 mbp chromosome (47 single base deletions; 15 single base additions and one base substitution). There were 6 changes in one plasmid and 1 change in another. The *C*. *taeniosporum* chromosome contains 3,264,813 bp; the plasmids 5,984, 69,744, and 163,055 bp.

### Coding sequence annotation

Initially, annotation of the chromosome was accomplished in four stages. The first stage was location of potential protein coding regions and identification of stable RNA coding sequences with RAST (http://rast.nmpdr.org/) [107] and the Institute for Genomic Sciences Annotation Engine (http://ae.igs.umaryland.edu/egi/index.cgi). Some individual (deduced) proteins were identified by Blast searches [108, 109] (www.ncbi.nlm.nih.gov/genomes/prokhits.cgi) against the NCBI Concise Protein Database. The second stage was to search deduced protein sequences for homology against the proteome of a closely related species, *C*. *botulinum* strain B1 Okra (ftp://ftp.ncbi.nlm.nih.gov/genomes/Bacteria/Clostridium_botulinum_B1_Okra_uid59147/NC_010516.faa). This data base is designated CbBO. The protein-to-protein BLASTp search of the BLAST+ package (http://www.ncbi.nlm.nih.gov/pubmed/20003500?dopt=Citation) was used. The third and fourth stages included searches against the bactNOG 4.1 database (http://eggnogdb.embl.de/download/eggnog_4.1/data/bactNOG/) and the Pfam protein domain database version 28.0 (http://pfam.xfam.org) [110]. HMMER 3.1 (http://www.hmmer.org) was used. Homology matches with an e-value greater than 1e-5 [14] were discarded. For each CDS, preference for the annotation was chosen in the order CbBO, bactNOG, and Pfam. That is, if no significant match was found in the CbBO database, the bactNOG database was search, and finally the Pfam database. The automated annotation was moderately curated manually.

Searches of the CbBO (ftp://ftp.ncbi.nlm.nih.gov/genomes/Bacteria/Clostridium_botulinum_B1_Okra_uid59147/NC_010516.ptt) and bactNOG databases also assigned CDSes into functional categories of clusters of orthologous groups (COGs); assignments were reviewed and edited manually with preference given to the CbBO assignment. The functional category list of prokaryotic proteins described by Tatusov et al. [111] was extended to include Phage-related proteins, Sporulation/control/appendages and Drug resistance/bacterial toxins (Table 1).

After the genome sequence was corrected by giving preference to the Illumina data, the annotation was updated by Genbank effective July 13, 2107 by the NCBI Prokaryotic Genome Annotation Pipeline and the manual curation repeated. The annotation uses GeneMarkS+ which incorporates both protein alignments and statistical predictions and is an extension of GeneMarkS [112, 113]. The update benefits from a combination of changes made in the corrected sequence and changes made by routine improvements to the annotation pipeline.

The replication origin, *oriC*, was found by Ori-Finder [19]. Similarity of (deduced) protein sequences was monitored with Lalign version 36.3.5e [114], internal repeat sequences were detected with Internal Repeats Finder [115], DUF11 was located with MyHits [116] and putative promoters located by Pattern Locator [117]. Prophage sequences were identified and annotated by PHAST (http://phast.wishartlab.com) [118].

### Prophage induction and electron microscopy

Prophage induction was accomplished by adding 5 μg ml^-1^ mitomycin C to exponentially growing cultures (absorbance 0.1) and incubating for four hours. The cultures were centrifuged at 24,000 g for 1 hr at 4°C and the supernatant filtered through 0.45 μm pore diameter Millipore filters. Polyethylene glycol 6000 was added to 10% (w/v) and dissolved. The preparation was incubated at 4°C for 60 min and centrifuged at 8000 g for 10 min at 4°C. The precipitated particles were resuspended in 0.01 times the original culture volume of deionized water, in place of the SM buffer used by Oakey and Owens [119]. The phages were observed by transmission electron microscopy. Ten microliters of 2% uranyl acetate were deposited on 200 mesh Formvar/Carbon coated copper grids. After 30 sec, 10 μl of phage preparation were mixed with the stain and, after 30 sec, the grids were gently blotted with Whatman paper and allowed to dry for 2–3 min. The grids were observed with an FEI Tecnai Spirit BioTwin transmission electron microscope operated at 80kV transmission electron microscope.

### Accession numbers

Sequences of the chromosome and the plasmids pCt1, pCt2 and pCt3 have been deposited in GenBank with Accession Numbers CP017253, CP017254, CP017255 and CP017256, respectively.

### Construction of pCt1-2200

A 2,397 bp fragment of pCt1 carrying a portion of the relaxase gene, the *rep* gene, the intergenic region downstream of *rep* and a portion of the amidopeptidase gene was amplified by PCR (forward primer, pCT1oriF1, 5’ GCAACTTAGAGAAGGCGAAAACCT; reverse primer, pCTori3p, 5’ GGTGGTAAAAACTCAGGCAAAATATCC) and cloned into pGem-T Easy Vector (Promega Corp. Madison, WI. 53711) (selecting for ampicillin-resistance in *E*. *coli*) generating pCt1-2010. An 1,197 bp fragment of pAT4 carrying the chloramphenicol-resistance (CM-R) determinant was amplified with primers constructed to contain ApaI, AatII and PstI sites upstream of the CM-R gene and with an SphI site downstream (forward primer pC194CMRF1, 5’ AGAGGAGGGCCCGACGTCCTGCAG- GCGCTTAAAACCAGTCATACCA; reverse primer pC194CMRR1, 5' AGAGGAGCATGCAGCCGACCATTCGACAAGTT). The amplified fragment was cut with ApaI and SphI and cloned into the pGem-T Easy Vector polylinker, generating pCt1-2011. The pGem-T Easy Vector region was deleted from pCt1-2011 by cutting with PstI on both sides, ligating the remaining fragment and transforming into *B*. *subtilis* strain SMY. The resulting plasmid, pCt1-2200, consists of only the pCt1 replicon and the CM-R gene.

## Supporting information

S1 Table *C*. *taeniosporum* genes associated with mobile genetic elements. (DOCX)

S2 Table *C*. *taeniosporum* stable RNA genes. (DOCX)

S3 Table *C*. *taeniosporum* spore appendage protein properties. (DOCX)

S4 Table *C*. *taeniosporum* prophage CtØ1 annotation. (DOCX)

S5 Table *C*. *taeniosporum* defective prophage CtdØ2 annotation. (DOCX)

S6 Table *C*. *taeniosporum* prophage CtØ3 annotation. (DOCX)

S7 Table *C*. *taeniosporum* plasmid pCt1 annotation. (DOCX)

S8 Table *C*. *taeniosporum* plasmid pCt2 annotation. (DOCX)

S9 Table *C*. *taeniosporum* plasmid pCt3 annotation. (DOCX)

## References

[1] Rode LJ, Slepecky RA. Bacterial spore appendages. CRC Crit Rev Microbiol. 1971;1: 1–27. 4114946 10.3109/10408417109104476

[2] Driks A. Surface appendages of bacterial spores. Mol Microbiol. 2007;63: 623–625. doi: 10.1111/j.1365-2958.2006.05564.x 17302795 10.1111/j.1365-2958.2006.05564.x

[3] Krasil'nikov NA, Duda VI, Pivovarov GE. Spore structure of two new species of anaerobic bacteria–*Clostridium taeniosporum* n. sp. and *Bacillus penicillus* n. sp. Mikrobiologiya. 1968;37: 488–493. Translation: Microbiology 1968;37: 395- 401. 5733247

[4] Rode LJ, Crawford MA, Williams MG. Clostridium spores with ribbon-like appendages. J Bacteriol. 1967;93: 1160–1173. 6025419 10.1128/jb.93.3.1160-1173.1967 PMC276565

[5] Yolton DP, Huettel RN, Simpson DK, Rode LJ. Isolation and partial chemical characterization of the spore appendages of *Clostridium taeniosporum*. J Bacteriol. 1972;109: 881–885. 5058456 10.1128/jb.109.2.881-885.1972 PMC285225

[6] Walker JR, Gnanam AJ, Blinkova AL, Hermandson MJ, Karymov MA, Lyubchenko YL, Graves PR, Haystead TA, Linse KD. *Clostridium taeniosporum* spore ribbon-like appendage structure, composition and genes. Mol Microbiol. 2007;63: 629–643. doi: 10.1111/j.1365-2958.2006.05494.x 17302797 10.1111/j.1365-2958.2006.05494.x

[7] Ramachandran GN, Doyle BB, Blout ER. Single-chain triple helical structure. Biopolymers. 1968;6: 1771–1775. doi: 10.1002/bip.1968.360061213 5704347 10.1002/bip.1968.360061213

[8] Beck K, Brodsky B. Supercoiled protein motifs: The collagen triple helix and the α-helical coiled coil. J Struct Biol. 1998;122: 17–29. doi: 10.1006/jsbi.1998.3965 9724603 10.1006/jsbi.1998.3965

[9] Emsley J, Knight CG, Farndale RW, Barnes MJ. Structure of the integrin α2β1- binding collagen peptide. J Mol Biol. 2004;335: 1019–1028. 14698296 10.1016/j.jmb.2003.11.030

[10] Shoulders MD, Raines RT. Collagen structure and stability. Annu Rev Biochem. 2009;78: 929–958. doi: 10.1146/annurev.biochem.77.032207.120833 19344236 10.1146/annurev.biochem.77.032207.120833 PMC2846778

[11] Iyer AV, Blinkova AL, Yang S-Y, Harrison M, Tepp WH, Jacobson MJ, et al *Clostridium taeniosporum* is a close relative of the *Clostridium botulinum* Group II. Anaerobe. 2008;14: 318–324. doi: 10.1016/j.anaerobe.2008.11.004 19135540 10.1016/j.anaerobe.2008.11.004 PMC5614447

[12] Gonchikov GG. Eukaryotes origin: a new scenario. J. Gen. Biol. 2010;71: 298–309. 20865931

[13] Krzywinski M, Schein J, Birol I, Connors J, Gascoyne R, Horsman D, et al Circos, an information aesthetic for comparative genomics. Genome Res. 2009;19: 1639–1645. doi: 10.1101/gr.092759.109 19541911 10.1101/gr.092759.109 PMC2752132

[14] Duan J, Jiang W, Cheng Z, Heikkila JJ, Glick BR. The complete genome sequence of the plant growth-promoting bacterium *Pseudomonas* sp. UW4. Plos One. 2013;8: e58640 doi: 10.1371/journal.pone.0058640 23516524 10.1371/journal.pone.0058640 PMC3596284

[15] Hennig S, Ziebuhr W. Characterization of the transposase encoded by IS*256*, the prototype of a major family of bacterial Insertion Sequence elements. J. Bacteriol. 2010;192: 4153–4163. doi: 10.1128/JB.00226-10 20543074 10.1128/JB.00226-10 PMC2916423

[16] Tormay P, Wilting R, Heider J, Bock A. Genes coding for the selenocysteine- inserting tRNA species from Desulfomicrobium baculatum and *Clostridium thermoaceticum*: Structural and evolutionary implications. J Bacteriol. 1994;176: 1268–1274. 8113164 10.1128/jb.176.5.1268-1274.1994 PMC205188

[17] Woese CR. Bacterial evolution. Microbiol Rev. 1987;51:221–271. 2439888 10.1128/mr.51.2.221-271.1987 PMC373105

[18] Ludwig W, Klenk H-P. Overview: A phylogenetic backbone and taxonomic framework for prokaryotic systematics In: Garrity GM, editor-in-chief. Bergey's manual of systematic bacteriology. 2^nd^ Ed Vol Two The Proteobacteria, Part A. Introductory Essays. New York, NY: Springer; 2005 pp. 49–66.

[19] Gao F, Zhang C-T. Ori-Finder: A web-based system for finding *oriC*s in unannotated bacterial genomes. BMC Bioinformatics. 2008;9: 79 doi: 10.1186/1471-2105-9-79 18237442 10.1186/1471-2105-9-79 PMC2275245

[20] Lemon KP, Moriya S, Ogasawara N, Grossman AD. Chromosome replication and segregation In: Sonenshein AL, Hoch JA, Losick R, editors. *Bacillus subtilis* and its closest relatives: from genes to cells. Washington, DC: American Society for Microbiology Press; 2002 pp. 73–86.

[21] Mott ML, Berger JM. DNA replication initiation: mechanisms and regulation in bacteria. Nature Rev. 2007;5: 343–354. 10.1038/nrmicro1640 17435790

[22] Noirot-Gros M-F, Polard P, Noirot P. Replication of the *Bacillus subtilis* chromosome In: Graumann P, editor. *Bacillus* Cellular & Molecular Biology. Norfolk, UK: Caister Academic Press; 2012 pp. 1–36.

[23] Moriya S, Atlung T, Hansen FG, Yoshikawa H, Ogasawara N. Cloning of an autonomously replicating sequence (*ars*) from the *Bacillus subtilis* chromosome. Mol Microbiol. 1992;6: 309–315. doi: 10.1111/j.1365-2958.1992.tb01473.x 1552845 10.1111/j.1365-2958.1992.tb01473.x

[24] Lobry JR. Asymmetric substitution patterns in the two DNA strands of bacteria. Mol Biol Evol. 1996;13: 660–665. 8676740 10.1093/oxfordjournals.molbev.a025626

[25] Lobry JR. Origin of replication of *Mycoplasma genitalium*. Science. 1996; 272: 745–746. 8614839 10.1126/science.272.5262.745

[26] Brewer BJ. Replication and the transcriptional organization of the *Escherichia coli* chromosome In: Drlica K, Riley M, editors. The Bacterial Chromosome. Washington, DC; American Society for Microbiology Press; 1990 pp. 61–83.

[27] Blattner FR, Plunkett G III, Bloch CA, Perna NT, Burland V, Riley M, et al The complete genome sequence of *Escherichia coli* K-12. Science. 1997;277: 1453–1462. 9278503 10.1126/science.277.5331.1453

[28] Freeman JM, Plasterer TN, Smith TF, Mohr SC. Patterns of genome organization in bacteria. Science. 1998;279: 1827a.

[29] Liu B, and Alberts BM. Head-on collision between DNA replication complex and RNA polymerase transcription complex. Science. 1995;267: 1131–1137. 7855590 10.1126/science.7855590

[30] Deshpande AM, and Newlon CS. DNA replication fork pause sites dependent on transcription.Science. 1996;272: 1030–1033. doi: 10.1126/science.272.5264.1030 8638128 10.1126/science.272.5264.1030

[31] Zhang Y, Turanov AA, Hatfield DL, Gladyshev VN. In silico identification of genes involved in selenium metabolism: evidence for a third selenium utilization trait. BMC Genomics. 2008;9: 251 doi: 10.1186/1471-2164-9-251 18510720 10.1186/1471-2164-9-251 PMC2432076

[32] Kogoma T. Origins of chromosome replication In: de Bruijn FJ, Lupski JR, Weinstock GM, editors. Bacterial Genomes Physical Structure and Analysis. New York, NY: Springer Science + Business Media; 1999 pp. 67–77.

[33] Briggs GS, Smits WK, Soultanas P. Chromosomal replication initiation machinery of low-G+C-content *Firmicutes*. J Bacteriol. 2012;194: 5162–5170. doi: 10.1128/JB.00865-12 22797751 10.1128/JB.00865-12 PMC3457243

[34] Salazar L, Fsihi H, de Rossi E, Riccardi G, Rios C, Cole ST, et al Organization of the origins of replication of the chromosomes of *Mycobacterium smegmatis*, *Mycobacterium leprae* and *Mycobacterium tuberculosis* and isolation of a functional origin from *M*. *smegmatis*. Mol Microbiol. 1996;20: 283–293. 8733228 10.1111/j.1365-2958.1996.tb02617.x

[35] Qin M-H, Madiraju MVVW, Zachariah S, Rajagopalan M. Characterization of the *oriC* region of *Mycobacterium smegmatis*. J Bacteriol. 1997;179: 6311–6317. 9335277 10.1128/jb.179.20.6311-6317.1997 PMC179544

[36] Qin M-H, Madiraju MVVW, Rajagopalan M. Characterization of the functional origin of *Mycobacterium tuberculosis*. Gene. 1999;233: 121–130. 10375628 10.1016/s0378-1119(99)00148-1

[37] Al-Hinai MA, Jones SW, Papoutsakis ET. The *Clostridium* sporulation programs: Diversity and preservation of endospore differentiation. Microbiol. Mol. Biol. Rev. 2015;79: 19–37. doi: 10.1128/MMBR.00025-14 25631287 10.1128/MMBR.00025-14 PMC4402964

[38] Collins MD, Lawson PA, Willems A, Cordoba JJ, Fernandez-Garayzabal J, Garcia P et al The phylogeny of the genus *Clostridium*: proposal of five new genera and eleven new species combinations. Internat. J. Sys. Bacteriol. 1994;44: 812–826. 10.1099/00207713-44-4-812 7981107

[39] Yutin N, Galperin MY. A genomic update on clostridial phylogeny: Gram-negative spore formers and other misplaced clostridia. Environ. Microbiol. 2013;15: 2631–2641. doi: 10.1111/1462-2920.12173 23834245 10.1111/1462-2920.12173 PMC4056668

[40] Collins MD, East AK. Phylogeny and taxonomy of the food-borne pathogen *Clostridium botulinum* and its neurotoxins. J. Appl. Microbiol. 1998;64: 5–17. 10.1046/j.1365-2672.1997.00313.x 15244052

[41] Iyer AV, Blinkova AL, Yang S-Y, Harrison M, Tepp WH, Jacobson MJ, Johnson EA, Bennett GN, Walker JR. *Clostridium taeniosporum* is a close relative of the *Clostridium botulinum* Group II. Anaerobe 2008;14: 318–324. doi: 10.1016/j.anaerobe.2008.11.004 19135540 10.1016/j.anaerobe.2008.11.004 PMC5614447

[42] Long S, Jones DT, Woods DR. The relationship between sporulation and solvent production in *Clostridium acetobutylicum* P262. Biotechnol. Lett. 1984;6: 529–534. doi: 10.1007/BF00139997

[43] Jones D, Van der Westhuizen A, Long S, Allcock E, Reid S, Woods D. Solvent production and morphological changes in *Clostridium acetobutylicum*. Appl. Environ. Microbiol. 1982;43: 1434–1439. 16346038 10.1128/aem.43.6.1434-1439.1982 PMC244251

[44] Kunkel B, Sandman K, Panzer S, Youngman P, Losick R. The promoter for a sporulation gene in the SpoIVC locus of *Bacillus subtilis* and its use in studies of temporal and spatial control of gene expression. J. Bacteriol. 1988;170: -3522. 10.1128/jb.170.8.3513-3522.1988 PMC211322 2841290

[45] Kirk DG, Dahlsten E, Zhang Z, Korkeala H, Lindstrom M. Involvement of *Clostridium botulinum* ATCC 3502 sigma factor K in early-stage sporulation. Appl. Environ. Microbiol. 2012;78: 4590–4596. doi: 10.1128/AEM.00304-12 22544236 10.1128/AEM.00304-12 PMC3370484

[46] Al-Hinai MA, Jones SW, Papoutsakis ET. SigmaK of *Clostridium acetobutylicum* is the first known sporulation-specific sigma factor with two developmentally separated roles, one early and one late in sporulation. J. Bacteriol. 2014;196: 287–299. doi: 10.1128/JB.01103-13 24187083 10.1128/JB.01103-13 PMC3911250

[47] Britton RA, Eichenberger P, Gonzalez-Pastor JE, Fawcett P, Monson R, Losick R, Grossman AD. Genome-wide analysis of the stationary-phase sigma factor (sigma-H) regulon of *Bacillus subtilis*. J. Bacteriol. 2002;184: 4881–4890. doi: 10.1128/JB.184.17.4881-4890.2002 12169614 10.1128/JB.184.17.4881-4890.2002 PMC135291

[48] Trach K, Burbulys D, Strauch M, Wu JJ, Dhillon N, Jonas R, et al Control of the initiation of sporulation in *Bacillus subtilis* by a phosphorelay. Res. Microbiol. 1991;142: 815–823. 1664534 10.1016/0923-2508(91)90060-n

[49] Dubnau E, Weir J, Nair G, Carter I, Moran C Jr, Smith I. *Bacillus* sporulation gene *spo0H* codes for sigma 30 (sigma H). J. Bacteriol. 1988;170: 1054–1062. 3277943 10.1128/jb.170.3.1054-1062.1988 PMC210873

[50] Dürre P. Sporulation in clostridia (genetics) In: Dürre P, editor. Handbook on clostridia. Boca Raton, FL; CRC Press; 2005 pp. 659–669.

[51] Mitchell A, Chang H-Y, Daugherty L, Fraser M, Hunter S, Lopez R, et al The InterPro protein families database: the classification resource after 15 years. Nucl Acids Res. 2015;43: D213–D221. doi: 10.1093/nar/gku1243 (http://www.ebi.ac.uk/interpro/entry/IPR001434). Accessed 5 February 2015. 25428371 10.1093/nar/gku1243 PMC4383996

[52] Marchler-Bauer A, Derbyshire MK, Gonzales NR, Lu S, Chitsaz F, Geer LY, et al CDD: NCBI's conserved domain database. Nucl Acids Res. 2015:43:D222–D226. doi: 10.1093/nar/gku1221 *(*http://www.ncbi.nlm.nih.gov/Structure/cdd/cdd.shtml). Accessed 9 December 2015. 25414356 10.1093/nar/gku1221 PMC4383992

[53] Reiter L, Tourasse NJ, Fouet AK, Loss R, Davison S, Økstad OA, et al Evolutionary history and functional characterization of three large genes involved in sporulation in *Bacillus cereus* group bacteria. J Bacteriol. 2011;193: 5420–5430. doi: 10.1128/JB.05309-11 21821775 10.1128/JB.05309-11 PMC3187416

[54] Birkelund S, Morgan-Fisher M, Timmerman E, Gevaert K, Shaw AC, Christiansen G. Analysis of proteins in *Chlamydia trachomatis* L2 outer membrane complex, COMC. FEMS Immunol Med Microbiol. 2009;55: 187–195. doi: 10.1111/j.1574-695X.2009.00522.x 19187221 10.1111/j.1574-695X.2009.00522.x

[55] Francoleon DR, Boontheung P, Yang Y, Kim U, Ytterberg AJ, Denny PA, et al S- layer, surface-accessible, and concanavalin A binding proteins of *Methanosarcina acetivorans* and *Methanosarcina mazei*. J Proteome Res. 2009;8: 1972–1982. doi: 10.1021/pr800923e 19228054 10.1021/pr800923e PMC2666069

[56] Xu Y, Keenes DR, Bujnicki JM, Höök M, Lukomski S. Streptococcal Scl1 and Scl2 proteins form collagen-like triple helices. J Biol Chem. 2002;277: 27312–27318. doi: 10.1074/jbc.M201163200 11976327 10.1074/jbc.M201163200

[57] Sylvestre P, Couture-Tose E, Mock M. Polymorphism in the collagen-like region of the *Bacillus anthracis* BclA protein leads to variation in exosporium filament length. J Bacteriol. 2003;185: 1555–1563. doi: 10.1128/JB.185.5.1555-1563.2003 12591872 10.1128/JB.185.5.1555-1563.2003 PMC148075

[58] Bamford DH, Bamford JKH. Collagenous proteins multiply. Nature 1990;344: 497. 10.1038/344497b0 2320123

[59] Engel J, Bächinger HP. Collagen-like sequences in phages and bacteria. Proc Indian Acad Sci (Chem Sci) 1999;111: 81–86.

[60] Wösten MMSM. Eubacterial sigma-factors. FEMS Microbiol Rev. 1998;22: 127–150. 9818380 10.1111/j.1574-6976.1998.tb00364.x

[61] Sierro N. Makita Y, de Hoon MJL, Nakai K. DBTBS: a database of transcriptional regulation in *Bacillus subtilis* containing upstream intergenic conservation. Nucl Acids Res. 2008;36 (Database issue): D93–D96. doi: 10.1093/nar/gkm910 (http://dbtbs.hgc.jp) Accessed 15 December 2015. 17962296 10.1093/nar/gkm910 PMC2247474

[62] Kawano M, Storz G, Rao BS, Rosner JL, Martin RG. Detection of low-level promoter activity within open reading frame sequences of *Escherichia coli*. Nucl Acids Res. 2005;33:6268–6276. doi: 10.1093/nar/gki928 16260475 10.1093/nar/gki928 PMC1275588

[63] Jackson EN, Yanofsky C. Internal promoter of the tryptophan operon of *Escherichia coli* is located in a structural gene. J Mol Biol. 1972;69: 307–313. 4560950 10.1016/0022-2836(72)90232-x

[64] Wek RC, Hatfield GW. Examination of the internal promoter, P_E_, in the *ilvGMEDA* operon of *E*. *coli* K-12. Nucl Acids Res. 1986;14: 2763–2777. 2421252 10.1093/nar/14.6.2763 PMC339697

[65] Maniloff J, Ackermann H-W. Taxonomy of bacterial viruses: establishment of tailed virus genera and the order *Caudovirales*. Arch Virol. 1998;143: 2051–2063. 9856093 10.1007/s007050050442

[66] Hatfull GF, Jacobs-Sera D, Lawrence JG, Pope WH, Russell DA, Ko C-C, et al Comparative genomic analysis of 60 mycobacteriophage genomes: Genome clustering, gene acquisition, and gene size. J Mol Biol. 2010;397: 119–143. doi: 10.1016/j.jmb.2010.01.011 20064525 10.1016/j.jmb.2010.01.011 PMC2830324

[67] Katsura I, Hendrix RW. Length determination in bacteriophage lambda tails. Cell 1984;39: 691–698. doi: 10.1016/0092-8674(84)90476-8 6096021 10.1016/0092-8674(84)90476-8

[68] Belcaid M, Bergeron A, Poisson G. The evolution of the tape measure protein: units, duplications and losses. BMC Bioinformatics 2011;12(Suppl 9): S10. 10.1186/1471-2105-12-S9-S10 PMC3271669 22151602

[69] Katoh K, Misawa K, Kuma K-I, Miyata T. MAFFT: a novel method for rapid multiple sequence alignment based on fast Fourier transform. Nucl Acids Res. 2002;30: 3059–3066. 12136088 10.1093/nar/gkf436 PMC135756

[70] Saltou N, Nei M. The neighbor-joining method: a new method for reconstructing phylogenetic trees. Mol Biol Evol. 1987;4: 406–425. 3447015 10.1093/oxfordjournals.molbev.a040454

[71] Mariotti M, Santesmasses D, Guigó R. Evolution of selenophosphate synthetase. In: Hatfield DL, Schweizer U, Tsuji PA, Gladyshev VN, editors. Selenium: Its Molecular Biology and Role in Human Health. Berlin/Heidelberg, Germany: Springer Science+Business Media; 2016 pp. 85–99. doi: 10.1007/978-3-319-41283–2_8

[72] Nisman B. The Stickland reaction. Bacteriol. Rev. 1954;18: 16–42. 13140081 10.1128/br.18.1.16-42.1954 PMC180783

[73] Turner DC, Stadtman TC. Purification of protein components of the clostridial glycine reductase system and characterization of protein A as a selenoprotein. Arch. Biochem. Biophys. 1973;154: 366–381 4734725 10.1016/0003-9861(73)90069-6

[74] Costilow RN. Selenium requirement for the growth of *Clostridium sporogenes* with glycine as the oxidant in Stickland reaction systems. J. Bacteriol. 1977;131: 366–368. 873891 10.1128/jb.131.1.366-368.1977 PMC235431

[75] Barker HA. Amino acid degradation by anaerobic bacteria. Annu. Rev. Biochem. 1981;50: 23–40. doi: 10.1146/annurev.bi.50.070181.000323 6791576 10.1146/annurev.bi.50.070181.000323

[76] Böck A. Biosynthesis of selenoproteins: an overview. Biofactors. 2000;11: 77–78. 10705967 10.1002/biof.5520110122

[77] Ching WM, Alzner-DeWeerd B, Stadtman TC. A selenium-containing nucleoside at the first position of the anticodon in seleno-tRNAGlu from *Clostridium sticklandii*. Proc. Natl. Acad. Sci. U.S.A. 1985;82: 347–350. 3918309 10.1073/pnas.82.2.347 PMC397035

[78] Wolfe MD, Ahmed F, Lacourciere GM, Lauhon CT, Stadtman TC, Larson TJ. ` Functional diversity of the rhodanese homology domain: the *Escherichia coli ybbB* gene encodes a seleno-phosphate-dependent tRNA 2-selenouridine synthase. J. Biol. Chem. 2004;279: 1801–1809. doi: 10.1074/jbc.M310442200 14594807 10.1074/jbc.M310442200

[79] Schräder T, Reinhöfer A, Andreesen JR. Selenium-containing xanthine dehydrogenase from *Eubacterium barkeri*. Eur. J. Biochem. 1999;264: 862–871. 10.1046/j.1432-1327.1999.00678.x 10491134

[80] Srivastava M, Mallard C, Barke T, Hancock LE, Self WT. A Selenium-dependent xanthine dehydrogenase triggers biofilm proliferation in *Enterococcus faecalis* through oxidant production. J. Bacteriol. 2000:2011; 1643–1652. doi: 10.1128/JB.01063-10 10.1128/JB.01063-10 PMC3067645 21257770

[81] Self WT, Wolfe MD, Stadtman TC. Cofactor determination and spectroscopic characterization of the selenium-dependent purine hydroxylase from *Clostridium purinolyticum*. Biochemistry. 2003;42: 11382–11390. doi: 10.1021/bi030136k 14503889 10.1021/bi030136k

[82] Self WT, Stadtman TC. Selenium-dependent metabolism of purines: a selenium- dependent purine hydroxylase and xanthine dehydrogenase were purified from Clostridium purinolyticum and characterized. Proc. Natl. Acad. Sci. U.S.A. 2000;97: 7208–7213. 10860985 10.1073/pnas.97.13.7208 PMC16524

[83] Dilworth GL. Occurrence of molybdenum in the nicotinic acid hydroxylase from *Clostridium barkeri*. Arch. Biochem. Biophys. 1983;221: 565–569. 6838209 10.1016/0003-9861(83)90176-5

[84] Gladyshev VN, Khangulov SV, Stadtman TC. Properties of the selenium- and molybdenum-containing nicotinic acid hydroxylase from *Clostridium barkeri*. Biochemistry. 1996;35: 212–223. doi: 10.1021/bi951793i 8555176 10.1021/bi951793i

[85] Myers GS, Rasko DA, Cheung JK, Ravel J, Seshadri R, DeBoy RT, et al Skewed genomic variability in strains of the toxigenic bacterial pathogen, *Clostridium perfringens*. Genome Res. 2006;16: 1031–1040. doi: 10.1101/gr.5238106 16825665 10.1101/gr.5238106 PMC1524862

[86] Stuknyte M, Guglielmetti S, Mora D, Kuisiene N, Parini C, Citavicius D. Complete nucleotide sequence of pGS18, a 62.8-kb plasmid from *Geobacillus stearothermophilus* strain 18. Extremophiles 2008;12: 415–429. doi: 10.1007/s00792-008-0145-y 18305896 10.1007/s00792-008-0145-y

[87] Murray NE. Type I restriction systems: Sophisticated molecular machines (a legacy of Bertani and Weigle). Microbiol Mol Biol Rev. 2000;64: 412–434. 10839821 10.1128/mmbr.64.2.412-434.2000 PMC98998

[88] Bruand C, Velten M, McGovern S, Marsin S, Sérèna C, Ehrlich SD, et al Functional interplay between the *Bacillus subtilis* DnaD and DnaB proteins essential for initiation and re-initiation of DNA replication. Mol Microbiol. 2005;55: 1138–1150. doi: 10.1111/j.1365-2958.2004.04451.x 15686560 10.1111/j.1365-2958.2004.04451.x

[89] Labri SJ, Samson JE, Moineau S. Bacteriophage resistance mechanisms. Nature Rev. 2010;8: 317–327. doi: 10.1038/nrmicro2315 20348932 10.1038/nrmicro2315

[90] Wilson GG, Murray NE. Restriction and modification systems. Annu Rev Genet. 1991;25: 585–627. doi: 10.1146/annurev.ge.25.120191.003101 1812816 10.1146/annurev.ge.25.120191.003101

[91] Barrangou R. Diversity of CRISPR-Cas immune systems and molecular machines. Genome Biol. 2015;16: 247 doi: 10.1186/s13059-015-0816-9 26549499 10.1186/s13059-015-0816-9 PMC4638107

[92] Melderen LV, Saavedra de Bast M. Bacterial toxin-antitoxin systems: more than selfish entities? PLoS Genet 2009;5: e1000437 doi: 10.1371/journal.pgen.1000437 19325885 10.1371/journal.pgen.1000437 PMC2654758

[93] Page R, Peti W. Toxin-antitoxin systems in bacterial growth arrest and persistence. Nature Chem Biol. 2016;12:208–214. doi: 10.1038/nchembiol.2044 26991085 10.1038/nchembio.2044

[94] Lehnherr H, Maguin E, Jafri S, Yarmolinsky MB. Plasmid addiction genes of bacteriophage P1; *doc*, which causes cell death on curing of prophage, and *phd*, which prevents host death when prophage is retained. J Mol Biol. 1993;233: 414–428. doi: 10.1006/jmbi.1993.1521 8411153 10.1006/jmbi.1993.1521

[95] Roy CR, Cherfils J. Structure and function of Fic proteins. Nature Rev. 2015;13: 631–640. doi: 10.1038/nrmicro3520 26299785 10.1038/nrmicro3520

[96] Mutschler H, Gebhardt M, Shoeman RL, Meinhart A. A Novel Mechanism of Programmed Cell Death in Bacteria by Toxin-Antitoxin Systems Corrupts Peptidoglycan Synthesis. PLoS Biol 2011;9: e1001033 doi: 10.1371/journal.pbio.1001033 21445328 10.1371/journal.pbio.1001033 PMC3062530

[97] Martin WF, Garg S, Zimorski V. Endosymbiotic theories for eukaryote origin. Phil. Trans. R. Soc. B Biol. Sci. 2015;370: 20140330 doi: 10.1098/rstb.2014.0330 10.1098/rstb.2014.0330 PMC4571569 26323761

[98] Baum DA. A comparison of autogenous theories for the origin of eukaryotic cells. American J. Bot. 2015;102: 1954–1965. doi: 10.3732/ajb.1500196 26643887 10.3732/ajb.1500196

[99] Atlas RM, Snyder JW. Handbook of media for clinical microbiology 2^nd^ ed Boca Raton: CRC Press Taylor & Francis Group; 2006 p. 118 & 226.

[100] Zeigler DR, Prágai Z, Rodriguez S, Chevreux B, Muffler A, Albert T, et al The origins of 168, W23, and other *Bacillus subtilis* legacy strains. J Bacteriol. 2008;190: 6983–6995. doi: 10.1128/JB.00722-08 18723616 10.1128/JB.00722-08 PMC2580678

[101] Horinouchi S, Weisblum B. Nucleotide sequence and functional map of pC194, a plasmid that specifies inducible chloramphenicol resistance. J Bacteriol. 1982;150:815–825. 6950931 10.1128/jb.150.2.815-825.1982 PMC216434

[102] Yasbin RE, Wilson GA, Young FE. Transformation and transfection in lysogenic strains of *Bacillus subtilis* 168. J Bacteriol. 1973;113: 540–548. 4632315 10.1128/jb.113.2.540-548.1973 PMC285263

[103] Sambrook J, Fritsch EF, Maniatis T. Molecular cloning: a laboratory manual 2nd ed Cold Spring Harbor, N.Y Cold Spring Harbor Laboratory Press; 1989.

[104] Sanger F, Nicklen S, Coulson AR. DNA sequencing with chain-terminating inhibitors. *Proc Natl Acad Sci USA* 1977;74: 5463–5467. doi: 10.1073/pnas.74.12.5463 271968 10.1073/pnas.74.12.5463 PMC431765

[105] Gordon D, Abajian C, Green P. Consed: a graphical tool for sequence finishing. Genome Res. 1998;8: 195–202. 9521923 10.1101/gr.8.3.195

[106] Luo C, Tsementzi D, Kyrpides N, Read T, Konstantinidis KT. Direct comparisons of Illumina vs Roche 454 sequencing technologies on the same microbial community DNA sample. PLoS One. 2012;7(2):e30087 doi: 10.1371/journal.pone.0030087 Epub 2012 Feb 10. Erratum in: PLoS One. 2012;7(3):10.1371/annotation/64ba358f-a483-46c2-b224-eaa5b9a33939. 22347999 10.1371/journal.pone.0030087 PMC3277595

[107] Aziz RK, Bartels D, Best AA, DeJongh M, Disz T, Edwards RA et al The RAST server: rapid annotations using subsystems technology. BMC Genomics 2008;9: 75 doi: 10.1186/1471-2164-9-75 18261238 10.1186/1471-2164-9-75 PMC2265698

[108] Altschul SF, Madden TL, Schaffer AA, Zhang J, Zhang Z, Miller W, et al Gapped BLAST and PSI-BLAST: a new generation of protein database search programs. Nucl Acids Res. 1997;25: 3389–3402. 9254694 10.1093/nar/25.17.3389 PMC146917

[109] Altschul SF, Wootton JC, Gertz EM, Agarwala R, Morgulis A, Schaffer AA, et al Protein database searches using compositionally adjusted substitution matrices. FEBS J. 2005;272: 5101–5109. doi: 10.1111/j.1742-4658.2005.04945.x 16218944 10.1111/j.1742-4658.2005.04945.x PMC1343503

[110] Finn RD, Bateman A, Clements J, Coggill P, Eberhardt RY, Eddy SR, et al Pfam: the protein families database. Nucl Acids Res. 2014;42: D222–D230. doi: 10.1093/nar/gkt1223 24288371 10.1093/nar/gkt1223 PMC3965110

[111] Tatusov RL, Galperin MY, Natale DA, Koonin EV. The COG database: a tool for genome-scale analysis of protein functions and evolution. Nucl Acids Res. 2000;28: 33–36. 10592175 10.1093/nar/28.1.33 PMC102395

[112] Besemer J, Lomsadze A, Rordovsky M. GeneMarkS: a self-training method for prediction of gene starts in microbial genomes. Implications for finding sequence motifs in regulatory regions. Nucl. Acids Res. 2001;29; 26-7–2618. 10.1093/nar/29.12.2607 PMC55746 11410670

[113] Tatusova T, DiCuccio M, Badretdin A, Chetvernin V, Ciufo S, Li W. Prokaryotic genome annotation pipeline In: The NCBI Handbook [Internet]. 2nd edition Bethesda, Md National Center for Biotechnology Information (US); 2013-. Available from: https://www.ncbi.nlm.nih.gov/books/NBK143764/

[114] Huang X, Miller W. A time-efficient, linear-space local similarity algorithm. Adv Appl Math. 1991;12: 337–357.

[115] Pellegrini M, Marcotte EM, Yeates TO. A fast algorithm for genome-wide analysis of proteins with repeated sequences. Proteins 1999;35: 440–446. 10382671

[116] Sigrist CJ, Cerutti L, de Castro E, Langendijk-Genevaux PS, Bulliard V, Bairoch A, et al PROSITE, a protein domain database for functional characterization and annotation. Nucl Acids Res. 2010;38(Database issue): D161–166. (http://myhits.isb-sib.ch/cgi-bin/motif_scan). Accessed 1 December 2015. doi: 10.1093/nar/gkp885 19858104 10.1093/nar/gkp885 PMC2808866

[117] Mrázek J, Xie S. Pattern locater: a new tool for finding local sequence patterns in genomic DNA sequences. Bioinformatics Applications Note 2006;22: 3099–3100. doi: 10.1093/bioinformatics/bt1551 http://www.cmbl.uga.edu/software.html. Accessed 22 December 2015. 10.1093/bioinformatics/btl551 17095514

[118] Zhou Y, Liang Y, Lynch KH, Dennis JJ, Wishart DS. PHAST: A fast phage search tool. Nucl Acids Res. 2011;39: Web Server Issue W347-W352. doi: 10.1093/nar/gkr485 10.1093/nar/gkr485 PMC3125810 21672955

[119] Oakey HJ, Owens L. A new bacteriophage, VHML, isolated from a toxin-producing strain of *Vibrio harveyi* in tropical Australia. J. Appl. Microbiol. 2000;89: 702–709. doi: 10.1046/j.1365-2672.2000.01169.x 11054176 10.1046/j.1365-2672.2000.01169.x

