# Supplementary material for: Genomics of Clostridium taeniosporum, an organism which forms endospores with ribbon-like appendages
Source: PLoS One. 2018 Jan 2;13(1):e0189673. doi: 10.1371/journal.pone.0189673 (PMC5749712; doi:10.1371/journal.pone.0189673)
Supplement: S1 Table — (DOCX) [file pone.0189673.s001.docx]

**Table S1. *C. taeniosporum* genes associated with mobile genetic elements.**

CDS CDS LOCATION PRODUCT

1. cmpl(39425..40651) IS256 family transposase

2. 482285..483487 transposase

3. 609002..610291 recombinase family protein

4. 610236..611566 recombinase family protein

5. cmpl(782838..784097) IS701 family transposase

6. cmpl(827955..829157) transposase

7. cmpl(860258..861484) IS256 family transposase

8. 891679..893289 IS5/IS1182 family transposase

9. 923502..923933 IS200/IS605 family transposase

10. 1244214..1245281 recombinase RecA

11. cmpl(1360859..1362076 IS701 family transposase

12. cmpl(1434892..1436013 transposase

13. 1493879..1495134 IS21 family transposase

14. 1495127..1495879 transposase

15. 1496042..1496959 integrase

16. cmpl(1608652..1610262 IS5/IS1182 family transposase

17. 1657216..1658205 tyrosine recombinase XerC

18. 1715383..1715733 transposase

19. 1715844..1717454 IS66 family transposase

20. 1751261..1752463 transposase

21. cmpl(1759006..1759437 IS200/IS605 family transposase

22. 1810054..1810260 IS30 family transposase

23. cmpl(1826325..1827551 IS256 family transposase

24. 1886855..1888081 IS256 family transposase

25. cmpl(1912435..1913661 IS256 family transposase

26. 1917983..1919209 IS256 family transposase

27. 1980753..1981970 IS701 family transposase

28. cmpl(1992199..1993426 IS256 family transposase

29. cmpl(2002741..2003619 recombinase XerD

30. cmpl(2008712..2009143 IS200/IS605 family transposase

31. cmpl(2011204..2012814 IS66 family transposase

32. cmpl(2012925..2013275 transposase

33. 2025844..2027139 IS21 family transposase

34. 2027132..2027884 transposase

35. cmpl(2028232..2029236 integrase

36. 2311470..2311820 transposase

37. 2311931..2313541 IS66 family transposase

38. 2328255..2328977 site-specific integrase

39. cmpl(2446833..2448050 IS701 family transposase

40. 2480631..2481779 transposase

41. 2481766..2483703 transposase

42. 2483693..2484079 transposase

43. cmpl(2504496..2504975 transposase

44. cmpl(2508813..2509199 transposase

45. cmpl(2509189..2511126 transposase

46. cmpl(2511113..2512261 transposase

47. cmpl(2514220..2514798 recombinase

48. 2518510..2519682 site-specific integrase

Table S1 continued.

49. cmpl(2534717..2535943 IS256 family transposase

50. 2546110..2547312 transposase

51. cmpl(2563754..2565364 IS5/IS1182 family transposase

52. cmpl(2625935..2627152 IS701 family transposase

53. cmpl(2756029..2756460 IS200/IS605 family transposase

54. cmpl(2869800..2870735 transposase

55. cmpl(2913503..2913934 IS200/IS605 family transposase

56. cmpl(2955302..2956528 IS256 family transposase

57. cmpl(3023467..3024693 IS256 family transposase

58. cmpl(3069074..3070105 transposase

59. cmpl(3100032..3101143 transposase

60. cmpl(3101121..3101708 IS607 family transposase

61. cmpl(3166742..3167944 transposase

62. cmpl(3241320..3242537 IS701 family transposase

cmpl,complement
