# Supplementary material for: Genomics of Clostridium taeniosporum, an organism which forms endospores with ribbon-like appendages
Source: PLoS One. 2018 Jan 2;13(1):e0189673. doi: 10.1371/journal.pone.0189673 (PMC5749712; doi:10.1371/journal.pone.0189673)
Supplement: S2 Table — (DOCX) [file pone.0189673.s002.docx]

**Table S2. *C. taeniosporum* stable RNA genes.**

CDS Location Product

1. 9859..11377 Small Subunit Ribosomal RNA; ssuRNA; SSU rRNA

2. 11598..14510 Large Subunit Ribosomal RNA; lsuRNA; LSU rRNA

3. 14562..14678 5S RNA

4. 14682..14758 tRNA-Met-CAT

5. 14763..14838 tRNA-Ala-TGC

6. 19467..19557 tRNA-Ser-TGA

7. 19592..19682 tRNA-Ser-GCT

8. 19913..20003 tRNA-Ser-TGA

9. 0038..20128 tRNA-Ser-GCT

10. 32909..32988 tRNA-Arg-ACG

11. 163874..163962 tRNA-Leu-TAA

12. 163983..164058 tRNA-Met-CAT

13. 164068..164144 tRNA-Met-CAT

14. 164149..164237 tRNA-Leu-TAA

15. 164258..164333 tRNA-Met-CAT

16. 164344..164420 tRNA-Met-CAT

17. 164426..164514 tRNA-Leu-TAA

18. 178464..178538 tRNA-Asn-GTT

19. 178857..178931 tRNA-Asn-GTT

20. 179252..179326 tRNA-Asn-GTT

21. 184194..185712 Small Subunit Ribosomal RNA; ssuRNA; SSU rRNA

22. 185831..185906 tRNA-Ala-TGC

23. 185910..185986 tRNA-Ile-GAT

24. 186096..189006 Large Subunit Ribosomal RNA; lsuRNA; LSU rRNA

25. 189058..189174 5S RNA

26. 189179..189254 tRNA-Phe-GAA

27. 189256..189329 tRNA-Cys-GCA

28. 215419..215494 tRNA-Thr-GGT

29. 393658..393733 tRNA-Pro-TGG

30. 393749..393822 tRNA-Gly-TCC

31. 394043..394119 tRNA-Arg-TCT

32. 394127..394202 tRNA-Pro-TGG

33. 394218..394291 tRNA-Gly-TCC

34. 394305..394381 tRNA-Arg-TCT

35. 394386..394461 tRNA-His-GTG

36. 394465..394539 tRNA-Gln-TTG

37. 394546..394621 tRNA-Lys-TTT

38. 394632..394716 tRNA-Leu-TAG

39. 394722..394796 tRNA-Gly-GCC

40. 394823..394896 tRNA-Gly-TCC

41. 394905..394980 tRNA-Lys-CTT

42. 394990..395065 tRNA-His-GTG

43. 395071..395145 tRNA-Gln-TTG

Table S2 continued.

44. 395152..395227 tRNA-Lys-TTT

45. 395238..395322 tRNA-Leu-TAG

46. 395328..395402 tRNA-Gly-GCC

47. 395429..395502 tRNA-Gly-TCC

48. 395543..395619 tRNA-Arg-TCG

49. 396907..398425 Small Subunit Ribosomal RNA; ssuRNA; SSU rRNA

50. 398542..398618 tRNA-Ile-GAT

51. 398888..401800 Large Subunit Ribosomal RNA; lsuRNA; LSU rRNA

52. 401852..401926 tRNA-Asn-GTT

53. 467442..467526 tRNA-Tyr-GTA

54. 467533..467608 tRNA-Val-TAC

55. 467633..467707 tRNA-Thr-TGT

56. 467715..467799 tRNA-Tyr-GTA

57. 467806..467881 tRNA-Val-TAC

58. 467907..467981 tRNA-Thr-TGT

59. 467987..468071 tRNA-Tyr-GTA

60. 494056..494130 tRNA-Trp-CCA

61. 500734..500808 tRNA-Trp-CCA

62. cmpl(1053762..1053837) tRNA-Met-CAT

63. (1612908..1612982) tRNA-Cys-GCA

64. cmpl(1816820..1816895) tRNA-Lys-TTT

65. cmpl(1816901..1816977) tRNA-Arg-TCT

66. cmpl(1819259..1819334) tRNA-Lys-TTT

67. cmpl(1819341..1819417) tRNA-Arg-TCT

68. cmpl(1819792..1819867) tRNA-Lys-TTT

69. cmpl(1819873..1819949) tRNA-Arg-TCT

70. cmpl(2259540..2259616) tRNA-Arg-TCT

71. cmpl(2280344..2280430) tRNA-Leu-GAG

72. 2331103..2331185 tRNA-Leu-CAA

73. 2478622..2478696 tRNA-Glu-CTC

74. cmpl(3021017..3021092) tRNA-Phe-GAA

75. cmpl(3021099..3021215) 5S RNA

76. cmpl(3040339..3040414) tRNA-Ala-TGC

77. cmpl(3040418..3040494) tRNA-Met-CAT

78. cmpl(3040503..3040619) 5S RNA

79. cmpl(3040721..3043633) Large Subunit Ribosomal RNA; lsuRNA; LSU rRNA

80. cmpl(3044135..3045653) Small Subunit Ribosomal RNA; ssuRNA; SSU rRNA

81. cmpl(3057640..3057756) 5S RNA

82. cmpl(3057874..3060786) Large Subunit Ribosomal RNA; lsuRNA; LSU rRNA

83. cmpl(3061291..3062809) Small Subunit Ribosomal RNA; ssuRNA; SSU rRNA

84. cmpl(3151477..3151568) tRNA-Ser-GGA

85. cmpl(3157405..3157479) tRNA-Arg-CCT

86. cmpl(3176349..3176465) 5S RNA

87. cmpl(3176583..3179495) Large Subunit Ribosomal RNA; lsuRNA; LSU rRNA

88. cmpl(3179715..3181233) Small Subunit Ribosomal RNA; ssuRNA; SSU rRNA

89. cmpl(3185804..3185879) tRNA-Lys-TTT

Table S2 continued

90. cmpl(3185887..3186003) 5S RNA

91. cmpl(3186966..3187041) tRNA-Lys-CTT

92. cmpl(3187048..3187164) 5S RNA

93. cmpl(3187336..3190248) Large Subunit Ribosomal RNA; lsuRNA; LSU rRNA

94. cmpl(3190587..3192105) Small Subunit Ribosomal RNA; ssuRNA; SSU rRNA

95. cmpl(3231917..3231993) tRNA-Asp-GTC

96. cmpl(3232008..3232083) tRNA-Val-TAC

97. cmpl(3232097..3232171) tRNA-Glu-TTC

98. cmpl(3232194..3232268) tRNA-Thr-TGT

99. cmpl(3232278..3232354) tRNA-Asp-GTC

100. cmpl(3232361..3232436) tRNA-Val-TAC

101. cmpl(3232449..3232523) tRNA-Glu-TTC

cmpl, complement
