# Supplementary material for: Genomics of Clostridium taeniosporum, an organism which forms endospores with ribbon-like appendages
Source: PLoS One. 2018 Jan 2;13(1):e0189673. doi: 10.1371/journal.pone.0189673 (PMC5749712; doi:10.1371/journal.pone.0189673)
Supplement: S3 Table — (DOCX) [file pone.0189673.s003.docx]

**Table S3. *C. taeniosporum* spore appendage protein properties.**

A. SIMILARITIES AMONG PROTEINS

PROTEIN TOTAL SIMILAR SIMILAR IDENTICAL SIMILAR E-VALUE

PAIRS LENGTH REGION REGION (%) (%)

(RESIDUES) (RESIDUES) LENGTH

(RESIDUES

PLUS GAPS)

P29a 269 1-269 269 87 95.9 1.2e-85

P29b 269 1-269

CRD1 564 163-556 399 36.6 68.7 2.7e-45

CRD2 413 13-410

P29a 269 13-240 233 25.3 60.9 1.2e-11

CRD1 564 38-269

P29a 269 19-238 225 21.8 59.6 4.4e-10

CRD1 564 175-399

P29b 269 12-240 233 24.9 58.8 1e-10

CRD1 564 37-269

P29b 269 19-238 225 22.2 59.6 3.4e-11

CRD1 564 175-399

P29a 269 8-258 258 29.8 59.7 1.1e-16

CRD2 413 17-272

P29b 269 8-258 258 29.1 58.1 1.2e-16

CRD2 413 17-272

HYPO2 158 20-156 140 34.3 65 8.7e-20

HYPO3 160 20-157

HYPO2 158 18-156 139 42.4 68.3 2.6e-21

CRD1 564 418-556

HYPO2 158 22-158 138 39.9 73.9 1.4e-23

CRD2 413 276-413

HYPO3 160 3-155 156 28.8 60.9 8.4e-11

CRD1 564 406-553

HYPO3 160 18-158 141 35.5 66 3e-18

CRD2 413 277-409

GP85 386 1-39 39 100 - 1.5e-15

CL2 307 1-39

GP85 386 40-216 179 51.4 58.1 8.6e-8

CL2 307 40-167

B. INTERNAL REPEAT AND DUF11 REGIONS

PROTEIN REPEATS DUF11

RESIDUES LENGTH IDENTITY E-VALUE DUF11 LENGTH E-VALUE

REPEATED (INCLUDES (%) RESIDUES (RESIDUES)

GAPS)

P29a 22-92 72 30.5 1.1e-6 23-56 34 6.5e-5

149-219 104-183 80 6.6e-5

P29b 22-92 72 26.3 1.1e-5 23-56 34 3e-4

149-219 104-183 80 5.3e-6

CRD1 141-266 127 25.9 2.5e-9 137-212 76 5.4e-4

275-399 279-343 65 1e-8

CRD2 16-119 104 26.9 2.1e-9 9-63 55 6e-5

148-250 142-195 54 8.3e-5

GP85 51-131 81 100 1e-0

125-205

CL2 45-109 65 ` 74.2 5.7e-14

103-165
