# Supplementary material for: Genomics of Clostridium taeniosporum, an organism which forms endospores with ribbon-like appendages
Source: PLoS One. 2018 Jan 2;13(1):e0189673. doi: 10.1371/journal.pone.0189673 (PMC5749712; doi:10.1371/journal.pone.0189673)
Supplement: S4 Table — (DOCX) [file pone.0189673.s004.docx]

**Table S4. *C. taeniosporum* prophage CtØ1 annotation.**

CDS POSITION BLAST HIT E-VALUE

576343..576354 attL TTTTAATATTAA N/A

577352..578488 PHAGE_Staphy_2638A_NC_007051: ORF010; PP_00517; phage(gi66395460) 7e-81

578536..579075 PHAGE_Crocei_P2559Y_NC_023614: hypothetical protein; PP_00518; phage(gi589893852) 1e-48

579145..581100 PHAGE_Strept_P9_NC_009819: polymerase; PP_00519; phage(gi157311150) 0.0

581169..583538 PHAGE_Staphy_3A_NC_007053: ORF002; PP_00520; phage(gi66395590) 0.0

583835..584122 PHAGE_Bacter_APSE_2_NC_011551: hypothetical protein; PP_00521; phage(gi212499740) 9e-17

584097..585476 PHAGE_Entero_phiFL4A_NC_013644: DNA helicase; PP_00522; phage(gi281416462) 6e-146

585478..585900 PHAGE_Strept_DCC1738_NC_024361: phage protein; PP_00523; phage(gi658307499) 1e-08

586111..586473 PHAGE_Bacill_phi105_NC_004167: hypothetical protein; PP_00524; phage(gi22855043) 9e-33

586533..586775 conserved protein of unknown function [*Tepidanaerobacter*

*acetatoxydans* Re1]. gi|438003893|ref|YP_007273636.1|; PP_00525 5e-25

586772..587152 PHAGE_Staphy_SPbeta_like_NC_029119: Fic/DOC family protein; PP_00526; phage(gi985761206) 3e-11

587372..587812 hypothetical protein CTC01932 [*Clostridium tetani* E88]. gi|28211561|ref|NP_782505.1|;

PP_00527 3e-82

587802..589025 PHAGE_Strept_EJ_1_NC_005294: transferase; PP_00528; phage(gi39653711) 6e-71

589094..589405 hypothetical protein CTC01929 [*Clostridium tetani* E88]. gi|28211559|ref|NP_782503.1|;

PP_00529 6e-47

589584..590489 PHAGE_Pseudo_RIO_1_NC_021300: amidoligase; PP_00530; phage(gi508178022) 9e-07

590558..590767 AIG2 family protein [*Clostridium tetani* 12124569]. gi|557606021|ref|YP_008774132.1|;

PP_00531 5e-31

590849..591331 PHAGE_Burkho_phi1026b_NC_005284: gp1; PP_00532; phage(gi38707891) 1e-18

591324..592871 PHAGE_Gordon_Zirinka_NC_031097:hypotheticalprotein;PP_00533;phage(gi100002) 3e-89 cmpl(592877..593515) hypothetical protein HPL003_22480 [*Paenibacillus terrae* HPL-003]. gi|374324314|ref|

YP_005077443.1|; PP_00534 6e-20

593722..594171 hypothetical; PP_00535 N/A

594309..595562 PHAGE_Entero_mEp235_NC_019708: portal protein; PP_00536; phage(gi428781813) 2e-72

595559..596245 PHAGE_Geobac_E2_NC_009552: putative Clp peptidase; PP_00537; phage(gi148747731) 4e-44

596259..597461 PHAGE_Entero_phiP27_NC_003356: putative major capsid protein; PP_00538;

phage(gi18249904) 6e-43

597485..597760 PHAGE_Bacill_phIS3501_NC_019502: phage DNA packaging protein; PP_00539; phage(gi422934332) 7e-06

597767..598093 PHAGE_Clostr_phiCT19406C_NC_029006: head-tail adaptor protein; PP_00540; phage(gi971821549)5e-25

598086..598472 PHAGE_Bacill_WBeta_NC_007734: conserved phage protein; PP_00541; phage(gi85701387) 1e-15

598805..599374 PHAGE_Bacill_WBeta_NC_007734: putative major tail protein; PP_00542; phage(gi85701389) 3e-33

599395..599682 PHAGE_Bacill_WBeta_NC_007734: conserved phage protein; PP_00543; phage(gi85701390) 3e-08

599900..602344 PHAGE_Geobac_E2_NC_009552: putative tail tape measure protein; PP_00544; phage(gi148747742)2e-71

602346..603053 PHAGE_Clostr_phiCT19406C_NC_029006: tail protein; PP_00545; phage(gi971821558) 9e-38

Table S4 continued.

603053..604909 PHAGE_Clostr_phiCT19406C_NC_029006: endopeptidase/tail protein; PP_00546;

phage(gi971821559) 2e-55 604911..606038 PHAGE_Clostr_phiCT19406A_NC_030950: hypothetical protein; PP_00547; phage(gi100058) 1e-09 606428..607771 PHAGE_Bacill_1_NC_009737: hypothetical protein; PP_00548; phage(gi155042949) 2e-20

607812..608219 PHAGE_Bacill_WBeta_NC_007734: phage holin; PP_00549; phage(gi85701395) 2e-26

608212..608916 PHAGE_Deep_s_D6E_NC_019544: lysin; PP_00550; phage(gi423262347) 2e-26

609002..610291 PHAGE_Bacill_WBeta_NC_007734: putative site-specific recombinase; PP_00551;

phage(gi85701406) 3e-37

610344..611285 PHAGE_Bacill_WBeta_NC_007734: putative site-specific recombinase; PP_00552;

phage(gi85701406) 3e-21 613755..613766 attR TTTTAATATTAA N/A

cmpl, complement
