# Supplementary material for: Genomics of Clostridium taeniosporum, an organism which forms endospores with ribbon-like appendages
Source: PLoS One. 2018 Jan 2;13(1):e0189673. doi: 10.1371/journal.pone.0189673 (PMC5749712; doi:10.1371/journal.pone.0189673)
Supplement: S5 Table — (DOCX) [file pone.0189673.s005.docx]

**Table S5*. C. taeniosporum* defective prophage CtdØ2 annotation.**

CDS POSITION BLAST HIT E VALUE

cmpl

(918585..919061) PHAGE_Clostr_phiCT9441A_NC_029022: hypothetical protein; PP_00835; phage(gi971821645) 2e-37

cmpl

(919066..919431) PHAGE_Thermo_THSA_485A_NC_018264: transcriptional regulator, XRE family; PP_00836;

phage(gi397912660) 3e-08

919739..920251 PHAGE_Clostr_PhiS63_NC_017978: gp40; PP_00837; phage(gi388570666) 5e-37

920781..921194 PHAGE_Brevib_Jimmer1_NC_029104: hypothetical protein; PP_00838; phage(gi985760809) 4e-13

921184..921339 PHAGE_Clostr_phiCT9441A_NC_029022: hypothetical protein; PP_00839; phage(gi971821692) 2e-08

921341..922651 PHAGE_Clostr_phiCT9441A_NC_029022: xkdK-like tail sheath protein; PP_00840;

phage(gi971821693) 5e-130

922670..923137 PHAGE_Brevib_Abouo_NC_029029: core tail protein; PP_00841; phage(gi971767182) 1e-52

923502..923933 PHAGE_Clostr_c_st_NC_007581: putative IS transposase (OrfA); PP_00842; phage(gi80159868) 3e-24

924116..924529 PHAGE_Clostr_phiCT9441A_NC_029022: xkdN-like protein; PP_00843; phage(gi971821696) 1e-48

924726..926639 PHAGE_Clostr_phiCT9441A_NC_029022: tail-tape measure protein; PP_00844;

phage(gi971821699) 9e-98

926639..927301 PHAGE_Clostr_phiCT9441A_NC_029022: xkdP-like LysM domain-containing protein; PP_00845; phage(gi971821700) 3e-73

927313..928284 PHAGE_Clostr_phiCT9441A_NC_029022: xkdP-like protein; PP_00846; phage(gi971821701) 8e-107

928288..928617 PHAGE_Clostr_phiCT9441A_NC_029022: hypothetical protein; PP_00847; phage(gi971821702) 3e-24

928610..929017 PHAGE_Clostr_phiCT9441A_NC_029022: xkdS-like protein; PP_00848; phage(gi971821703) 2e-38

929020..930078 PHAGE_Clostr_phiCT9441A_NC_029022: Cro/CI family transcriptional regulator; PP_00849; phage(gi971821704) 2e-95

930079..933132 PHAGE_Clostr_phiCT9441A_NC_029022: CopG family transcriptional regulator; PP_00850; phage(gi971821705) 2e-36 933134..933751 PHAGE_Clostr_phiCT9441A_NC_029022: portal protein; PP_00851; phage(gi971821708) 3e-60

933766..934776 PHAGE_Clostr_phiCT9441A_NC_029022: hypothetical protein; PP_00852; phage(gi971821709) 8e-07

934790..935182 hypothetical protein Cspa_c07590 [*Clostridium saccharoperbutylacetonicum* N1-4(HMT)]. gi|451817589|ref|YP_007453790.1|; PP_00853 3e-23 935172..935321 hypothetical; PP_00854 N/A

935424..935624 UviB-like protein [*Clostridium botulinum* E3 str. Alaska E43].

gi|188588691|ref|YP_001920299.1|; PP_00855 1e-25

935640..935870 PHAGE_Clostr_phiCP39_O_NC_011318: holin; PP_00856; phage(gi208429883) 1e-12

935936..936700 PHAGE_Clostr_phi24R_NC_019523: N-acetylmuramoyl-L-alanine amidase; PP_00857;

phage(gi422936723) 7e-44

Table S5 Continued.

936868..937458 PHAGE_Pseudo_Lu11_NC_017972: hypothetical protein; PP_00858; phage(gi388684814) 2e-05

937651..938697 PHAGE_Bacill_SPbeta_NC_001884: hypothetical protein; PP_00859; phage(gi9630153) 3e-09

938716..939753 hypothetical protein CLL_A0972 [*Clostridium botulinum* B str. Eklund 17B (NRP)]. gi|187935393|ref|YP_001885173.1|; PP_00860 2e-164

939905..941014 PHAGE_Bacill_G_NC_023719: gp245; PP_00861; phage(gi593777701) 7e-31

cmpl, complement
