# Supplementary material for: Genomics of Clostridium taeniosporum, an organism which forms endospores with ribbon-like appendages
Source: PLoS One. 2018 Jan 2;13(1):e0189673. doi: 10.1371/journal.pone.0189673 (PMC5749712; doi:10.1371/journal.pone.0189673)
Supplement: S6 Table — (DOCX) [file pone.0189673.s006.docx]

**Table S6*. C. taeniosporum* prophage CtØ3 annotation.**

CDS LOCATION BLAST HIT E VALUE

2475831..2475842 attL AATTACTTATAG N/A

2476582..2476595 attL CTTTAATTTTCTTT N/A

2478509..2478520 attL CTTTAATATTCA N/A

2480646..2481779 PHAGE_Strept_phiBHN167_NC_022791: phage integrase; PP_02264; phage(gi557745680) 3e-09

2481766..2483703 PHAGE_Strept_PH15_NC_010945: putative integrase; PP_02265; phage(gi190151416) 2e-06

2483693..2484079 hypothetical protein PTH_2449 [*Pelotomaculum thermopropionicum* SI]. gi|147678784|ref|YP_001212999.1|; PP_02266 6e-29

cmpl(2484177..2484455) PHAGE_Clostr_phiCT453B_NC_029004: hypothetical protein; PP_02267;

2484674..2484880 PHAGE_Clostr_phiCT453B_NC_029004: hypothetical protein; PP_02268; phage(gi100060) 2e-06

cmpl(2484964..2485704) PHAGE_Clostr_phi8074_B1_NC_019924: endolysin CS74L; PP_02269; phage(gi431810380) 2e-33

cmpl(2485747..2486166) PHAGE_Clostr_vB_CpeS_CP51_NC_021325: putative holin; PP_02270; phage(gi509140062) 6e-47

cmpl(2486265..2486561) PHAGE_Clostr_phiCD211_NC_029048: hypothetical protein; PP_02271; phage(gi981220886) 3e-05

cmpl(2486594..2486968) hypothetical protein Cspa_c07590 [*Clostridium saccharoperbutylacetonicum* N1-4(HMT)].

gi|451817589|ref|YP_007453790.1|; PP_02272 1e-27

cmpl(2486980..2490363) PHAGE_Clostr_phiCD111_NC_028905: Tail fiber protein; PP_02273; phage(gi971820065) 3e-08

cmpl(2490382..2492229) PHAGE_Lactob_PLE2_NC_031036: hypothetical protein; PP_02274; phage(gi100017) 6e-32

cmpl(2492226..2492894) PHAGE_Clostr_phiCT19406C_NC_029006: tail protein; PP_02275; phage(gi971821558) 3e-19

cmpl(2492905..2495643) PHAGE_Clostr_phiCT453B_NC_029004: hypothetical protein; PP_02276; phage(gi100045) 2e-95

cmpl(2495648..2495953) hypothetical protein lse_1601 [*Listeria seeligeri* serovar 1/2b str. SLCC3954].

gi|289434966|ref|YP_003464838.1|; PP_02277 6e-13

cmpl(2495956..2496255) hypothetical; PP_02278 N/A

cmpl(2496256..2496819) hypothetical protein lse_1603 [*Listeria seeligeri* serovar 1/2b str. SLCC3954].

gi|289434968|ref|YP_003464840.1|; PP_02279 6e-49

cmpl(2496819..2497172) hypothetical protein lse_1604 [*Listeria seeligeri* serovar 1/2b str. SLCC3954].

gi|289434969|ref|YP_003464841.1|; PP_02280 5e-09

cmpl(2497173..2497583) PHAGE_Entero_phiFL4A_NC_013644: hypothetical protein; PP_02281; phage(gi281416477) 7e-20

cmpl(2497576..2497890) hypothetical; PP_02282 N/A

cmpl(2497895..2498212) PHAGE_Lactoc_PLgT_1_NC_031016: hypothetical protein; PP_02283; phage(gi100021) 2e-05

cmpl(2498251..2499138) PHAGE_Bacill_phi4J1_NC_029008: major capsid protein; PP_02284; phage(gi971821638) 2e-75 cmpl(2499158..2499766) PHAGE_Clostr_phiCDHM19_NC_028996: putative scaffold protein; PP_02285; phage(gi971763873) 4e-21

cmpl(2499939..2500106) hypothetical; PP_02286 N/A

cmpl(2500109..2501617) PHAGE_Clostr_phiCD119_NC_007917: putative head protein; PP_02287; phage(gi90592642) 2e-18

cmpl(2501607..2503076) PHAGE_Staphy_CNPx_NC_031241: hypothetical protein; PP_02288; phage(gi100003) 9e-77

cmpl(2503103..2504503) phage protein [*Lachnoclostridium phytofermentans* ISDg]. gi|160881099|ref|YP_001560067.1|; PP_02289 0.0

cmpl(2504496..2504975) PHAGE_Lactoc_98201_NC_031064: terminase small subunit; PP_02290; phage(gi100030) 1e-41

Table S6 continued.

cmpl(2505032..2505547) hypothetical protein Tola_1120 [*Tolumonas auensis* DSM 9187]. gi|237807892|ref|YP_002892332.1|; PP_02291 1e-14

cmpl(2505966..2506181) hypothetical; PP_02292 N/A

cmpl(2506184..2506708) PHAGE_Clostr_phiCT453B_NC_029004: siderophore-interacting protein; PP_02293; phage(gi100025) 3e-12

cmpl(2506710..2506889) hypothetical protein CLL_A1930 [*Clostridium botulinum* B str. Eklund 17B (NRP)].

gi|187934451|ref|YP_001886123.1|; PP_02294 3e-10

cmpl(2506893..2507210) hypothetical protein Cbei_3280 [*Clostridium beijerinckii* NCIMB 8052]. gi|150018111|ref|YP_001310365.1|; PP_02295 8e-12

cmpl(2507210..2507461) PHAGE_Clostr_vB_CpeS_CP51_NC_021325: putative stage III sporulation protein D; PP_02296; phage(gi509140082) 4e-13

cmpl(2507549..2507749) hypothetical protein CLL_A1932 [*Clostridium botulinum* B str. Eklund 17B (NRP)]. gi|187933195|ref|YP_001886125.1|; PP_02297 5e-26

cmpl(2507762..2508163) PHAGE_Clostr_phi3626_NC_003524: putative single stranded DNA binding protein; PP_02298; phage(gi20066002) 1e-21

2508180..2508191 attR CTTTAATATTCA N/A

cmpl(2508182..2508577) PHAGE_Clostr_phiCD27_NC_011398: putative endodeoxyribonuclease; PP_02299; phage(gi209901305) 2e-21

cmpl(2508813..2509199) hypothetical protein PTH_2449 [Pelotomaculum thermopropionicum SI]. gi|147678784|ref|YP_001212999.1|; PP_02300 6e-29

cmpl(2509189..2511126) PHAGE_Strept_PH15_NC_010945: putative integrase; PP_02301; phage(gi190151416) 2e-06 cmpl(2511113..2512246) PHAGE_Strept_phiBHN167_NC_022791: phage integrase; PP_02302; phage(gi557745680) 3e-09

cmpl(2512399..2512719) hypothetical; PP_02303 N/A

cmpl(2512723..2512893) hypothetical; PP_02304 N/A

cmpl(2512933..2513436) hypothetical protein CLL_A2773 [*Clostridium botulinum* B str. Eklund 17B (NRP)].

gi|187935130|ref|YP_001886961.1|; PP_02305 5e-45 cmpl(2513466..2514209) PHAGE_Lister_vB_LmoS_293_NC_028929: DnaD domain protein; PP_02306; phage(gi971756925) 2e-49

cmpl(2514220..2514798) PHAGE_Staphy_Slt_NC_002661: hypothetical protein; PP_02307; phage(gi12719409) 1e-37

cmpl(2514811..2515323) PHAGE_Staphy_80_NC_030652: RecT family recombinase; PP_02308; phage(gi100012) 4e-24

cmpl(2515337..2515528) hypothetical protein CLL_A1943 [*Clostridium botulinum* B str. Eklund 17B (NRP)].

gi|187933285|ref|YP_001886136.1|; PP_02309 1e-15

cmpl(2515587..2515757) hypothetical protein CLL_A1945 [*Clostridium botulinum* B str. Eklund 17B (NRP)].

gi|187933077|ref|YP_001886138.1|; PP_02310 3e-12

cmpl(2515732..2515944) PHAGE_Clostr_phiCT19406A_NC_030950: hypothetical protein; PP_02311; phage(gi100010) 3e-07

cmpl(2515962..2516792) PHAGE_Clostr_CDMH1_NC_024144: putative anti-repressor protein; PP_02312; phage(gi640884902) 2e-37

cmpl(2516817..2517059) excisionase family [*Clostridium saccharoperbutylacetonicum* N1-4(HMT)].

gi|451819473|ref|YP_007455674.1|; PP_02313 9e-20

cmpl(2517059..2517271) PHAGE_Thermo_THSA_485A_NC_018264: helix-turn-helix domain protein; PP_02314; phage(gi397912659) 3e-12

2517403..2517416 attR CTTTAATTTTCTTT N/A

2517496..2518008 PHAGE_Thermo_THSA_485A_NC_018264: transcriptional regulator, XRE family; PP_02315; phage(gi397912660) 7e-11

2518022..2518495 PHAGE_Clostr_phiCT19406A_NC_030950: hypothetical protein; PP_02316; phage(gi100004) 2e-30

2518510..2519682 PHAGE_Clostr_phiMMP01_NC_028883: putative integrase; PP_02317; phage(gi971820006) 9e-34

2519734..2519745 attR AATTACTTATAG N/A

cmpl, complement
