# Supplementary material for: Genomics of Clostridium taeniosporum, an organism which forms endospores with ribbon-like appendages
Source: PLoS One. 2018 Jan 2;13(1):e0189673. doi: 10.1371/journal.pone.0189673 (PMC5749712; doi:10.1371/journal.pone.0189673)
Supplement: S7 Table — (DOCX) [file pone.0189673.s007.docx]

Table S7 *C. taeniosporum* plasmid pCt1 annotation.

Functional

CDS Location Product E value Category Database

106..912 relaxase/Mobilization nuclease domain protein 4.0e-08 L bactNOG

1026..2258 replication protein 6.5e-64 L IGS

cmpl(2683..3681) peptidase 1.0e-18 R CbBO

cmpl(3772..4140) Bacterial SH3 domain 4.4e-10 S Pfam

4279..4782 Protein of unknown function (DUF536) 8.4e-06 S Pfam

5224..5433 Bacterial mobilisation protein (MobC) 7.7e-10 L Pfam

cmpl, complement
