# Supplementary material for: Genomics of Clostridium taeniosporum, an organism which forms endospores with ribbon-like appendages
Source: PLoS One. 2018 Jan 2;13(1):e0189673. doi: 10.1371/journal.pone.0189673 (PMC5749712; doi:10.1371/journal.pone.0189673)
Supplement: S8 Table — (DOCX) [file pone.0189673.s008.docx]

Table S8. *C. taeniosporum* plasmid pCt2 annotation

CDS Location Product E value FC Database

205..558 XRE family transcription regulator 7.0e-07 K CbBO

560..1384 metallo-endopeptidase (DUF955) 8.0e-106 R bactNOG

1401..2078 hypothetical protein - S -

cmpl(2227..4980) DNA repair helicase Rad25 1.0e-05 L CbBO

5678..6097 hypothetical protein - S -

6160..6330 YvrJ protein family 4.8e-18 S Pfam

cmpl(6420..6719) hypothetical protein - S -

cmpl(6813..7565) sporulation initiation inhibitor protein soj 1.0e-34 W CbBO

8182..8727 Iron-sulfur cluster-binding domain 1.9e-07 R Pfam

8742..9020 Coenzyme PQQ synthesis protein D (PqqD) 7.3e-09 R Pfam

9025..9669 ABC transporter ATP-binding protein 1.0e-39 R CbBO

cmpl(9964..10431) Acetyltransferase (GNAT) domain 7.5e-08 R Pfam

10937..11176 hypothetical protein - S -

cmpl(12187..12807) haloacid dehalogenase 3.0e-11 R CbBO

cmpl(13114..13647) Protein of unknown function (DUF2247) 3.6e-33 S Pfam

cmpl(13817..14131) hypothetical protein - S -

cmpl(14098..14430) hypothetical protein - S -

cmpl(14563..14892) hypothetical protein - S -

cmpl(14907..15959) hypothetical protein - S -

cmpl(15976..16419) hypothetical protein - S -

cmpl(16891..18225) replication protein 6.2e-60 L IGS

cmpl(19180..20538) MATE efflux family protein 3.0e-25 X CbBO

cmpl(21429..22232) hypothetical protein - S -

cmpl(22229..22930) ABC-2 family transporter protein 5.3e-15 R Pfam

cmpl(22932..23825) bacitracin ABC transporter ATP-binding protein BcrA 8.0e-99 X CbBO

cmpl(24559..24939) Retroviral aspartyl protease 1.0e-47 O bactNOG

cmpl(24939..25184) hypothetical protein - S -

cmpl(25467..26174) hypothetical protein - S -

26445..26987 Aminoglycoside N6-acetyltransferase 6.0e-51 X bactNOG

27518..28123 RNA polymerase sigma factor σ^70^ family 4.0e-16 K CbBO

29330..29437 Bacteriocin class II with double-glycine leader peptide 9.3e-06 * Pfam

29555..29839 Enterocin A Immunity 5.4e-18 * Pfam

30729..31457 accessory gene regulator AgrB 2.0e-10 K CbBO

31728..33041 sensor histidine kinase GHKL domain 2.0e-53 T CbBO

33144..33857 LytTr family DNA-binding response regulator 5.0e-80 T CbBO

Table S8 continued.

34595..34963 hypothetical protein - -

35000..37198 bacteriocin-associated integral membrane protein 0.0e+00 * bactNOG

37201..37817 ABC transporter ATP-binding protein 2.0e-39 R CbBO

37879..39312 Subtilase family 2.2e-54 O Pfam

cmpl(39606..41216) hypothetical protein - S -

cmpl(41295..42251) C-5 cytosine-specific DNA methylase 1.0e-33 L CbBO

44125..44361 Protein of unknown function (DUF1659) 2.2e-15 S Pfam

44402..44623 Protein of unknown function (DUF2922) 8.0e-15 S Pfam

cmpl(45509..45952) hypothetical protein - S -

cmpl(45966..46328) Nucleotidyltransferase domain 7.9e-09 R Pfam

cmpl(46577..47641) DNA replication protein DnaD 5.0e-06 L CbBO

cmpl(49024..49827) cell wall binding repeat domain protein 2.7e-22 M IGS

cmpl(49867..50607) hypothetical protein - S -

cmpl(51059..52297) DNA replication protein DnaD 4.0e-07 L CbBO

52997..53815 hypothetical protein - S -

cmpl(53891..55162) putative phage lysozyme 4.7e-26 V IGS

55715..56386 Protein of unknown function (DUF1311) 3.0e-71 S bactNOG

cmpl(57220..58431) DNA replication protein DnaD 7.0e-07 L CbBO

59001..59390 Cro/C1-type HTH DNA-binding domain transcription regulator 2.7e-08 R Pfam

59402..60196 Protein of unknown function (DUF955) 1.8e-09 S Pfam

60183..61067 hypothetical protein - S -

cmpl(61467..62651) Type I restriction modification DNA specificity domain 5.5e-23 L Pfam

cmpl(62665..64209) Type I restriction-modification system, M subunit 8.4e-129 L IGS

64484..65719 Abortive infection protein 7.0e-113 ** bactNOG

65703..66362 RloB-like protein 4.5e-39 L Pfam

cmpl(67555..68141) Sau1hsdS1restriction endonuclease subunit S 2.5e-16 L IGS

cmpl(68154..69698) Type I restriction-modification system, M subunit 6.9e-130 L IGS

* Bacteriocin-related genes; ** Bacteriophage-resistance gene; cmpl, complement
