# Supplementary material for: Genomics of Clostridium taeniosporum, an organism which forms endospores with ribbon-like appendages
Source: PLoS One. 2018 Jan 2;13(1):e0189673. doi: 10.1371/journal.pone.0189673 (PMC5749712; doi:10.1371/journal.pone.0189673)
Supplement: S9 Table — (DOCX) [file pone.0189673.s009.docx]

Table S9 *C. taeniosporum* plasmid pCt3 annotation.

CDS LOCATION PRODUCT E VALUE FC Database

cmpl(508..3681) Restriction Endonuclease subunit R 0.0e+00 L bactNOG

cmpl(3694..5643) site specific DNA methyltransferase 1.7e-35 L Pfam

cmpl(5734..5970) hypothetical protein - -

cmpl(6735..6977) hypothetical protein - -

cmpl(7064..7765) hypothetical protein - -

8343..8964 CRISPR-like direct repeat region

cmpl(9697..10341) hypothetical protein - -

cmpl(10893..11051) putative lipoprotein 1.0e-11 M IGS

11557..12162 RNA polymerase sigma factor σ^70^ family 1.0e-15 K CbBO

cmpl(12197..13708) clostripain 0.0e+00 X CbBO

14491..15525 exopolysaccharide biosynthesis protein 6.0e-47 M Pfam

15543..16571 dipeptidyl peptidase IV O

cmpl(17210..18130) site-specific tyrosine recombinase XerC 4.0e-117 L CbBO

18361..18897 nitroreductase 1.0e-82 R CbBO

cmpl(19840..20772) Protein of unknown function (DUF4163) 3.6e-07 S Pfam

21877..22401 acetyltransferase 4.0e-14 R CbBO

cmpl(22634..23023) hypothetical protein - -

cmpl(23017..23769) sporulation initiation inhibitor protein soj ParA family 2.0e-37 B CbBO

cmpl(24049..24291) prevent-host-death family 2.0e-22 X bactNOG

24906..25142 Protein of unknown function (DUF1659) 1.1e-13 S Pfam

25183..25401 Protein of unknown function (DUF2922) 7.2e-18 S Pfam

cmpl(25949..27817) cadmium-translocating P-type ATPase 0.0e+00 P CbBO

cmpl(27827..28048) cadmium-translocating P-type ATPase 5.0e-08 P CbBO

cmpl(28556..29545) AraC family transcriptional regulator 4.0e-70 K CbBO

cmpl(29726..31096) MATE efflux family protein 4.0e-143 X CbBO

cmpl(31611..32249) thiamine-phosphate pyrophosphorylase 2.0e-67 H CbBO

cmpl(32487..33317) hydroxyethylthiazole kinase 3.0e-65 H CbBO

cmpl(33320..34636) thiamine biosynthesis protein ThiC 0.0e+00 H CbBO

cmpl(34897..35703) phosphomethylpyrimidine kinase 6.0e-98 H CbBO

cmpl(35835..36353) thiW protein energy coupling factor 5.0e-24 H CbBO

cmpl(36543..36653) thiamine pyropohosphate – TPP riboswitch

37218..37514 hypothetical protein - -

cmpl(37661..38569) hypothetical protein - -

38740..38967 hypothetical protein - -

39370..40365 serine protease 3.0e-20 O CbBO

Table S9 continued.

41213..41743 signal peptidase I 1.0e-28 U CbBO

cmpl(42843..43943) hypothetical protein - -

44895..45170 pseudo gene

45305..46234 hypothetical protein - -

Cmpl(46561..46695) pseudo gene

49121..49648) hypothetical protein - -

cmpl(49664..50155) hypothetical protein - -

cmpl(50215..50826) hypothetical protein - -

cmpl(50847..51215) hypothetical protein - -

cmpl(51215..51478) hypothetical protein - -

cmpl(51478..51789) hypothetical protein - -

cmpl(51912..52439) hypothetical protein - -

cmpl(52436..52615) hypothetical protein - -

cmpl(52805..53050) pseudo gene

cmpl(53563..55077 phage late control gene D protein 1.7e-08 V Pfam

cmpl(55110..55487) Protein of unknown function (DUF4280) 9.5e-29 S Pfam

cmpl(55576..56103) hypothetical protein - -

cmpl(56234..56836) hypothetical protein - -

cmpl(56977..58401) Phage tail protein 3.7e-08 V Pfam

cmpl(58401..58994) hypothetical protein - -

cmpl(59575..61041) hypothetical protein - -

61297..61575 hypothetical protein - -

cmpl(61860..62516) vanZF protein 6.0e-06 X CbBO

63197..64165 patatin-like phospholipase 1.0e-106 I CbBO

cmpl(64566..64874) Protein of unknown function (DUF3795) 1.4e-10 S Pfam

65210..65875 hypothetical protein - -

66353..66592 hypothetical protein - -

cmpl(66605..67084) hypothetical protein - -

cmpl(67194..67640) Acetyltransferase (GNAT) family 4.0e-11 R Pfam

cmpl(67701..68174) AraC family transcription regulator - K -

cmpl(68225..68851) hypothetical protein - -

69327..69509 hypothetical protein - -

Cmpl(69557..69721) pseudo gene

cmpl(69770..70261) DNA mismatch repair protein MutT 1.7e-78 L IGS

cmpl(70429..70809) hypothetical protein - -

cmpl(71445..72101) radical SAM domain-containing protein 2.0e-12 R CbBO

cmpl(73060..73938) phosphoesterase 7.3e-109 R IGS

cmpl(74206..74628) hypothetical protein - -

Table S9 continued.

cmpl(75011..75598) Cupin 2, conserved barrel domain protein 4.0e-91 R bactNOG

cmpl(75877..76194) hydrolase, alpha/beta fold family protein 2.8e-49 R IGS

cmpl(76307..77254) hypothetical protein - -

cmpl(77257..77592) PadR-family transcriptional regulator 1.0e-50 K CbBO

cmpl(77957..79020) hypothetical protein - -

cmpl(79215..80195) Alpha Beta Hydrolase Fold protein 0.0e+00 R bactNOG

cmpl(80229..81158) putative phosphotransferase enzyme family 1.7e-149 R IGS

81574..81819 hypothetical protein - -

cmpl(82005..83099) hypothetical protein - -

cmpl(83517..84563) D-galactose-binding periplasmic protein 6.7e-137 G IGS

cmpl(84841..85269) N-acetyltransferase 4.0e-37 R CbBO

cmpl(85375..85680) hypothetical protein - -

cmpl(85802..86770) hypothetical protein - -

cmpl(86776..87099) ParR family transcription regulator

87327..89006 AAA family ATPase 3.7e-74 R Pfam

cmpl(89241..89810) hypothetical protein - -

cmpl(90015..90807) pseudo gene

cmpl(91235..91930) hypothetical protein - -

92407..93042 hypothetical protein - -

93418..94746 hypothetical protein - -

95060..96625 DNA mismatch repair MutS protein 0.0e+00 L CbBO

96664..98145 Dolichyl-phosphate-mannose-protein

mannosyltransferase 1.0e-17 G Pfam

98670..99020 CGGC domain 7.8e-22 S Pfam

99077..100087 iron chelate uptake ABC transporter

solute-binding protein 6.0e-35 P CbBO

100144..101178 iron ABC transporter permease 2.0e-112 P CbBO

101178..101951 iron ABC transporter ATP-binding protein 2.0e-88 P CbBO

102190..102354 YvrJ protein family 2.9e-18 Pfam

cmpl(102522..104544) pseudo gene

104782..105537 Endonuclease/Exonuclease/phosphatase family 2.0e-10 L Pfam

cmpl(105740..106348) Protein of unknown function (DUF1062) 2.0e-40 S bactNOG

cmpl(106615..106833) pseudo gene - -

106995..107495 nitroreductase 1.0e-92 C CbBO

cmpl(107791..108648) AraC family transcriptional regulator 0.0e+00 K CbBO

cmpl(108906..109550) TetR family transcriptional regulator 2.0e-07 K CbBO

109706..111367 EmrB/QacA family drug resistance transporter 3.0e-49 X CbBO

111405..111878 hypothetical protein - -

Table S9 continued.

112042..112770 endonuclease I 4.0e-07 L bactNOG

113336..113983 DNA-binding response regulator 5.0e-47 T CbBO

113973..115346 sensor histidine kinase 6.0e-30 T CbBO

115383..117683 putative lipoprotein 0.0e+00 M IGS

cmpl(117928..119595) thioredoxin-disulfide reductase 5.0e-102 O CbBO

cmpl(119700..120263) alkyl hydroperoxide reductase 2.0e-125 P bactNOG

120784..121563 Putative phosphatase regulatory subunit 2.0e-17 R bactNOG

cmpl(121680..122765) NlpC/p60-like transpeptidase 1.6e-64 O Pfam

cmpl(122843..123658) MerR family transcriptional regulator 1.0e-65 K CbBO

cmpl(124292..125044) proline iminopeptidase 2.0e-07 O CbBO

cmpl(125237..126847) Transposase 9.0e-144 L bactNOG

cmpl(126958..127308) Transposase IS66 family 5.0e-11 L bactNOG

cmpl(127296..127628) hypothetical protein - -

cmpl(127772..128023) hypothetical protein - -

cmpl(128424..128834) peptide deformylase 9.0e-11 O CbBO

cmpl(129296..130105) MATE efflux family protein 4.0e-24 X CbBO

cmpl(130176..130697) cysteine hydrolase 3.0e-06 O CbBO

cmpl(130867..132162) ATP-dependent RNA helicase RhlE 0.0e+00 K CbBO

cmpl(132397..133440) Fic/DOC family 1.1e-18 D Pfam

cmpl(133616..134629) polysaccharide deacetylase 2.0e-77 M CbBO

cmpl(134862..136012) Protein of unknown function 1.0e-151 bactNOG

cmpl(136298..136690) pseudo gene

cmpl(136702..137451) ABC-2 family transporter protein 2.5e-09 R Pfam

cmpl(137448..138248) bacitracin transport ATP-binding protein BcrA 2.0e-58 X CbBO

138755..140317 AAA family ATPase R

cmpl(140532..141179) chloramphenicol acetyltransferase 4.0e-52 X CbBO

141657..142313 Protein of unknown function (DUF1311) 4.0e-70 S bactNOG

cmpl(142417..143631) sodium:dicarboxylate symporter family protein 5.0e-27 P CbBO

144507..144716 hypothetical protein 3.0e-06 S bactNOG

cmpl(144882..145628) proline iminopeptidase 5.0e-09 O CbBO

cmpl(146690..147445) N-acetyltransferase (GNAT) family 2.7e-07 R Pfam

cmpl(147849..148652) putative lipoprotein 5.3e-107 M IGS

cmpl(150446..150670) hypothetical protein

151041..152054 integral membrane protein domain protein 1.9e-14 R IGS

152474..153842 O-antigen ligase 4.0e-103 M bactNOG

cmpl(154446..155018) Zeta toxin 1.0e-84 X bactNOG

cmpl(155024..155197) hypothetical protein - -

cmpl(156518..157069) hypothetical protein - -

Table S9 continued.

cmpl(157659..158132) hypothetical protein - -

cmpl(158128..158334) Protein of unknown function (DUF3990) 3.1e-18 S Pfam

cmpl(158428..159054) DJ-1/PfpI family protein 1.0e-133 R CbBO

cmpl(159458..160375) transcriptional regulator 0.0e+00 K CbBO

cmpl(160581..162191) Transposase 9.0e-144 L bactNOG

cmpl(162302..162502) ISPsy5 Orf1 5.0e-11 S bactNOG

cmpl(162640..162972) hypothetical protein - -

cmpl, complement
